# Chiral platinum (II)-4-(2,3-dihydroxypropyl)- formamide oxo-aporphine (FOA) complexes promote tumor cells apoptosis by directly targeting G-quadruplex DNA *in vitro* and *in vivo*

**DOI:** 10.18632/oncotarget.18778

**Published:** 2017-06-28

**Authors:** Qi-Pin Qin, Jiao-Lan Qin, Ming Chen, Yu-Lan Li, Ting Meng, Jie Zhou, Hong Liang, Zhen-Feng Chen

**Affiliations:** ^1^ State Key Laboratory for Chemistry and Molecular Engineering of Medicinal Resources, School of Chemistry and Pharmacy, Guangxi Normal University, Guilin 541004, P. R. China

**Keywords:** chiral platinum(II) complex, oxoaporphine, G-quadruplex DNA, telomerase, antitumor activity

## Abstract

Three platinum(II) complexes, 4 (LC-004), 5 (LC-005), and 6 (LC-006), with the chiral FOA ligands R/S-(±)-FOA (1), R-(+)-FOA (2) and S-(–)-FOA (3), respectively, were synthesized and characterized. As potential anti-tumor agents, these complexes show higher cytotoxicity to BEL-7404 cells than the HL-7702 normal cells. They are potential telomerase inhibitors that target c-myc and human telomeric G-quadruplex DNA. Compared to complexes 4 and 5, 6 exhibited higher binding affinities towards telomeric, c-myc G-quadruplex DNA and caspase-3/9, thereby inducing senescence and apoptosis to a greater extent in tumor cells. Moreover, our *in vivo* studies showed that complex 6 can effectively inhibit tumor growth in the BEL-7404 and BEL-7402 xenograft mouse models and is less toxic than 5-fluorouracil and cisplatin. The effective inhibition of tumor growth is attributed to its interactions with 53BP1, TRF1, c-myc, TRF2, and hTERT. Thus, complex 6 can serve as a novel lead compound and a potential drug candidate for anticancer chemotherapy.

## INTRODUCTION

**Figure 1:**
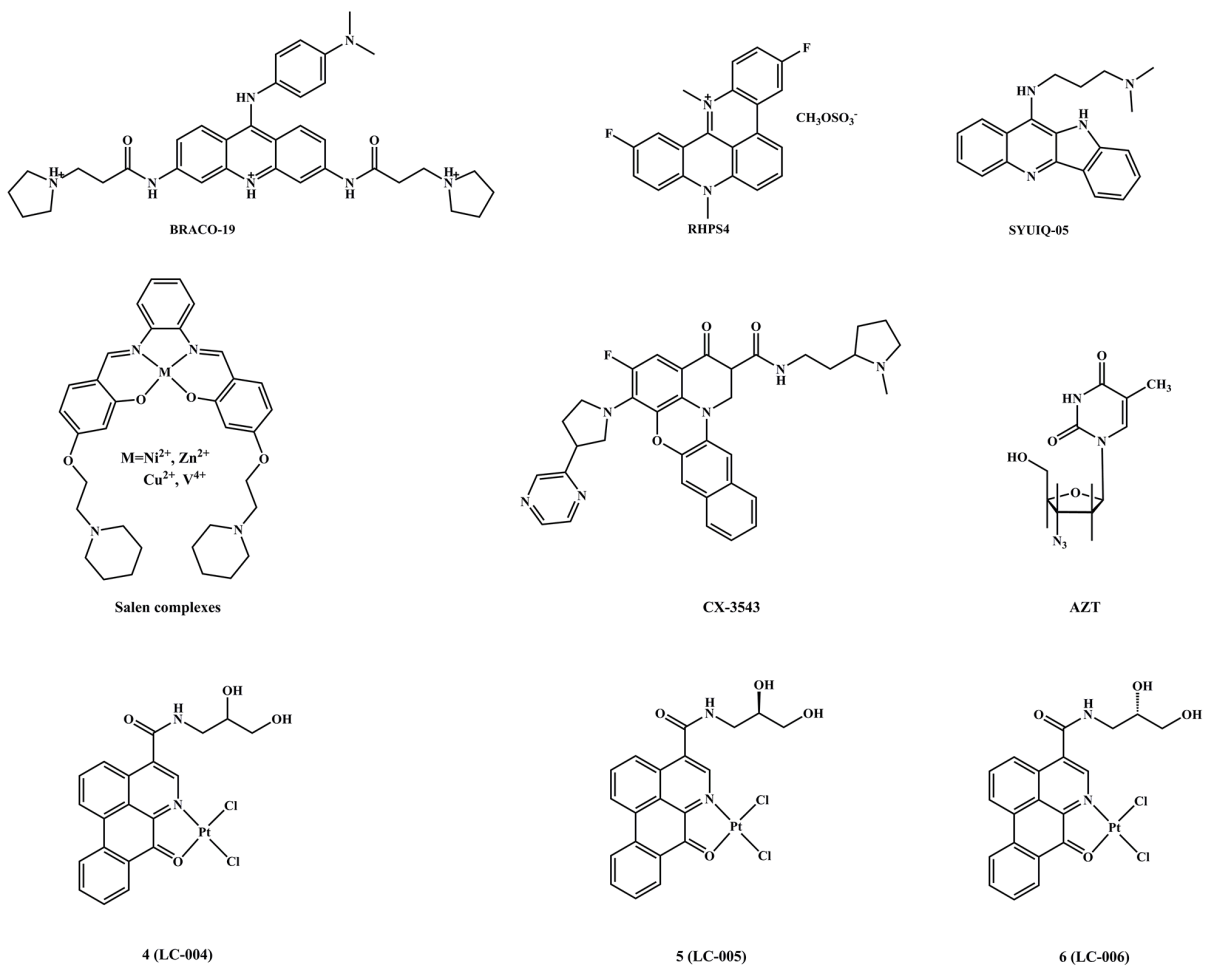
The structures of G4-DNA binders and telomerase inhibitors

Targeting G4s is currently considered as a practical strategy to design new anticancer drugs [1, 2]. It has been reported that many diseases, such as cancer [3], HIV [4], and diabetes [5], are closely related to G4s structures, which widely exist in variety of genes including bcl-2, c-myc, k-ras, the telomeres and c-kit G-quadruplex-forming sequences, and plays a crucial role in regulating the gene expression of different oncogenes [6–12]. Compared to DNA G-quadruplexes, little is known about RNA G-quadruplexes which are also considered as potential targets for the development of anticancer drugs [13–16]. Several studies have reported c-myc/G4s is a key activator for the expression of hTERT, which has been shown to play an important role in cell apoptosis/growth and senescence [17, 18]. C-myc G4 and/or other G4s could inhibit telomerase activity because the single-stranded RNA of the telomerase complex does not recognize G4 DNA [17, 18]. Therefore, it is not surprising that most of the available atomic-resolution level insights into G-quadruplex DNA-ligand interactions are related to c-myc G4 (Pu27 G-quadruplex) which is a potential therapeutic target [17]. In addition, recent studies show that some G4 ligands can effectively stabilize the G4 structure and cause inhibition of telomerase [19–24]. In this regard, a number of organic compounds and metal complexes, such as the acridine derivative BRACO19 [25, 26], RHPS4 [27], the quindoline derivative SYUIQ-05 [28, 29], salen complexes [30, 31], phthalocyanine [32], Ru(II) polypyridyl complexes [33], CX-3543 [34] and AZT [35, 36], have been designed to target G4s and telomerase [23, 24] (Figure 1). However, little is known about their anticancer activities *in vivo* and their detailed mechanisms of actions.

Over the last decades, platinum(II)-based drugs were widely used in anticancer chemotherapies. Some representative drugs are carboplatin, cisplatin, and oxaliplatin, which bind to double-strand DNA and disrupt DNA replication and transcription. However, all these platinum-based agents are associated with drug resistance, high toxicity *in vivo* and severe side effects [37, 38]. Thus, it was important to study and develop less toxic, more effective, and target-specific Pt-based anticancer drugs, such as a G-quadruplex ligand and telomerase inhibitors [6–12, 39–44]. Up to date, a number of anticancer platinum(II) agents targeting G4-DNA and telomerase have been explored [45–51], such as 4,4’-bpy platinum supramolecular square [52], platinum(II) phenylpehnanthroimidazole [53], propeller-shape trinuclear Pt(II) complexes [54] and porphyrin-bridged tetranuclear Pt(II) clovers [55]. In addition, some chiral antitumor platinum(II) complexes have been exploited [56–60], such as [PtCl_2_(R-(+)-BINAP)_2_], [PtCl_2_(S-(–)-BINAP)_2_], [PtCl_2_(R-(+)-DABN)_2_] and [PtCl_2_(S-(–)-DABN)_2_] (BINAP= 2,2’-bis(diphenylphosphane)-1,10-binaphthyl and DABN= 1,1’-binaphthyl-2,2’-diamine), which are aromatic diamines and aromatic bisphosphanes. Generally, the R-(+) configurations are less cytotoxic to cancer cell lines and less likely to interact with the nucleobases of the human telomeric G-quadruplex than those of the S-(–) isomer [61, 62]. However, very few exhibit superior binding affinities to G4-DNA [63]. Therefore, there is an unmet need to develop platinum complexes with higher anticancer activities *in vivo* and *in vitro*.

To date, several small enantiomer molecules display strong DNA binding activities [64], especially S-(–) isomer, such as [PtCl_2_L_2_] [65, 66], and Pt(II) drug oxaliplatin [67], which exhibit remarkable tumor growth inhibition and high selectively stabilized G4s as well as cellular uptake. Previously, we designed three chiral Ru(II) complexes with the FOA ligands R/S-(±)-FOA (1), R-(+)-FOA (2) and S-(–)-FOA (3) as anticancer agents [68]. In this study, three chiral platinum(II) complexes: 4 (LC-004) cis-[PtCl_2_(R/S-(±)-FOA)], 5 (LC-005) cis-[PtCl_2_(R-(+)-FOA)] and 6 (LC-006) cis-[PtCl_2_(S-(–)-FOA)] (Figure 1 and Supplementary Figure 1) were synthesized by employed three types of FOA ligands. Among these platinum(II) complexes, we found complex 6 exhibited higher selectivity and telomerase inhibition via targeting telomere G4s in BEL-7404 cells, as well as inducing S phase arrest and telomeres/DNA damage, which resulted in cell senescence and apoptosis. Importantly, through *in vivo* studies we demonstrated that complex 6 has high capacity to inhibit tumor growth, while less toxicity to normal cells, which further indicated the functional potential of complex 6 as a promising drug candidate for anticancer chemotherapy.

## RESULTS

### Synthesis and characterization of the chiral platinum(II) complexes

Three chiral ligands were synthesized and purified according to the method reported previously [68]. Complexes 4, 5 and 6 were synthesized as illustrated in Supplementary Figure 1 and characterized by CD spectroscopy, elemental analyses, IR spectroscopy, ESI-MS,^1^H and ^13^C NMR spectroscopy (Supplementary Figures 1–14). Based on the analytical and spectroscopic results, the molecular structures of complexes 4, 5, and 6 are determined as 4-coordinated square-planar geometry with ligands from FOA and two chlorines (Figure 1 and Supplementary Figure 1).

We next determined the solubility and stability of complexes 4, 5, and 6 in H_2_O and TBS buffer by UV-vis spectroscopy [68, 69]. Our data showed that the solubility of these three complexes reached 0.68, 1.00 and 2.00 mg/mL in water (Supplementary Figure 15), respectively. TBS buffer (1% DMSO, 100 mM KCl, and 10 mM pH 7.35 Tris-HCl) was used to mimic normal physiological conditions. No obvious changes in the absorption peaks and shapes for the complexes 4–6 over the time (24 h) were observed, demonstrating that complexes 4, 5, and 6 were table in their coordinating mode in TBS solution (Supplementary Figure 16). Furthermore, the retention times for complexes 4–6 remained unchanged under the same condition (mobile phase: 88:12 methanol/H_2_O) by HPLC experiments for a 24 h duration, further suggesting they were also stable enough in DMSO stock solution (Supplementary Figure 17).

### Evaluation of the cytotoxicity, cellular uptake and cellular distribution of chiral platinum(II) complexes

**Figure 2:**
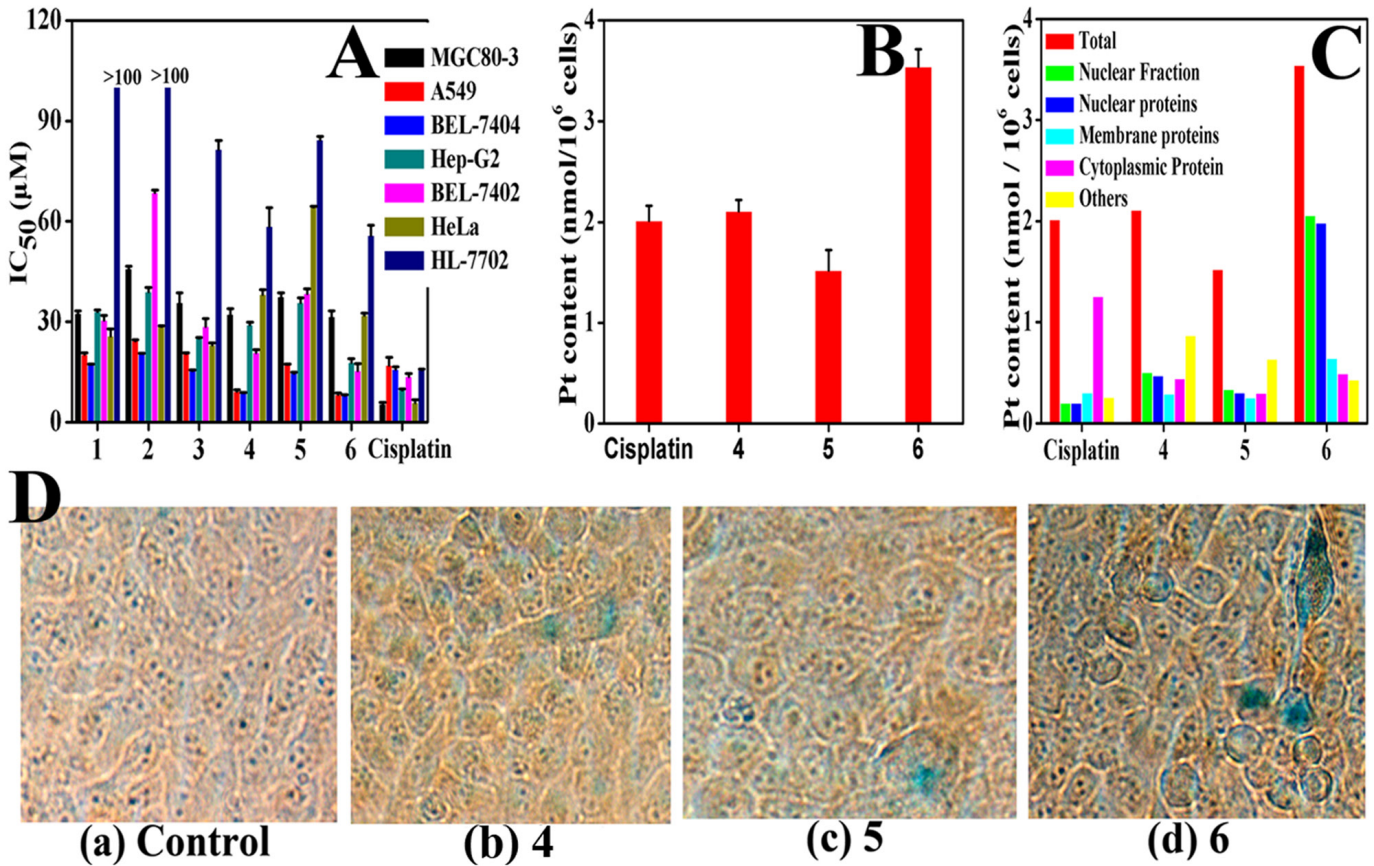
Complexes 4–6 induced cell senescence **(A)** Complexes 1–6 and cisplatin towards six cancer-cell lines and one HL-7702 cell line for 48 h. **(B)** and **(C)** Complexes 4–6 (10 *μ*M) treated of BEL-7404 cells for 8.0 h at 37 °C, comparing with cisplatin (10 *μ*M), respectively. Pt content in whole cell (B) and in different fraction (C) were measured by ICP-MS. **(D)** Complexes 4–6 (2.0 *μ*M) induce cell senescence in BEL-7404 cells: these cells were treatment of complexes 4–6 with 2.0 *μ*M for 7d, and using β-galactosidase stain were examined by fluorescence microscopy (Nikon Te2000 microscope, 200×).

To evaluate the cytotoxicity of chiral platinum(II) complexes, HeLa, BEL-7402, MGC80-3, BEL-7404, A549, Hep-G2 and HL-7702 cells (normal cells) were treated with varying concentrations of complexes 1–6 and cisplatin (positive control, cisplatin was dissolved at a concentration of 1.0 mM in 0.154 M NaCl) for 24 h and 48 h. The cell viability of each experimental group was examined by MTT assays. As shown in Figure 2A and Supplementary Tables 2–5, complexes 4–6 exhibited higher cellular inhibition in all cell lines except the HeLa cell line, compared to their corresponding ligands 1–3 [68]. As evident from the Supplementary Table 2–5 and Figure 2A, complexes 4–6 exhibited smaller IC_50_ values than their corresponding ligands 1–3 in all cell lines but the HeLa cell line. The BEL-7404 cell lines showed the highest sensitivity to complexes 4–6 with IC_50_ values of 12.5 ± 1.1, 22.5 ± 1.3 and 10.1 ± 0.6 *μ*M, and 8.7 ± 0.1, 14.5 ± 0.5, and 7.9 ± 0.3 *μ*M at 24 h and 48 h, respectively. The IC_50_ value differences between complexes 4–6 interaction of 24 h and 48 h are little, and the variation trend is similar. The significantly improved cytotoxicity behavior by complex 6 may be correlated with S-(–)-FOA ligand, similar to previous reported results of [PtCl_2_(S-(–)-BINAP)_2_] and [PtCl_2_(S-(–)-DABN)_2_] [61, 62]. Importantly, the cytotoxicities of complexes 4–6 tested in the present work between BEL-7404 tumor cells and normal HL-7702 cells were characterized by remarkably high the selectivity index factors [70], more than 3.0 (Supplementary Tables 4 and 5); Among these complexes, complex 6 demonstrated the highest selectivity towards BEL-7404 tumor cells at 24 h with SI (the selectivity index factor) was 11.8, which was 1.1 folds higher than these cancer cells treatment with complex 6 at 48 h (SI= 10.7). Therefore, complex 6 (10 μM) was selected for cell apoptosis analysis by flow-cytometry, RT-PCR, caspase-3/9 activation assays, western blot and transfection assays in BEL-7404 cells for 24 h. In addition, this observed difference in *in vitro* cytotoxicity between the three complexes 4–6 could be due to the influence of the chirality of the FOA ligand [68]. Nonetheless, these IC_50_ values are smaller than the corresponding IC_50_ value of cisplatin (15.8 ± 0.7 *μ*M or 24.8 ± 1.8 *μ*M), suggesting that these three compounds have higher cytotoxicity to BEL-7404 cells than cisplatin, especially with complex 6 treated cells. Notably, in normal cells (HL-7702 cells), complexes 4–6 displayed higher IC_50_ values compared to cisplatin, indicating that these three compounds are less toxic to normal human cells than cisplatin.

The cellular uptake of clinically relevant compounds usually affects their bioactivity [71]. We next explored the cellular intake and distribution of complexes 4–6 by using BEL-7404 cell line, because complexes 4–6 exhibited the highest *in vitro* cytotoxicity in this cell line compared to other cancer cell lines. ICP-MS was used to quantitate the cellular level of platinum [72]. As shown in Figure 2B, the level of platinum (Pt) in complexes 4–6 treated cells was significantly increased compared with cisplatin treated cells. Remarkably, the Pt concentration in complex 6 treated cells was 1.7, 2.3, and 2.5 times higher than that of complexes 4, 5 and cisplatin treatment group, respectively.

Furthermore, the distributions of cisplatin and complexes 4–6 in BEL-7404 cells were measured using the method reported by Chen and Schreiber [68, 73]. The results indicated that complex 6 was highly accumulated in nuclear fraction (including nuclear proteins), while complexes 4, 5, and cisplatin were mainly accumulated in cytoplasmic proteins and other cellular fractions (Figure 2C). Together, the observation of differences subcellular distribution of cisplatin and complexes 4–6 was consistent with the apoptotic pathways upon short-time exposure that these compounds activated.

### Chiral platinum(II) complexes induce BEL-7404 cell senescence and apoptosis *in vitro*

**Figure 3:**
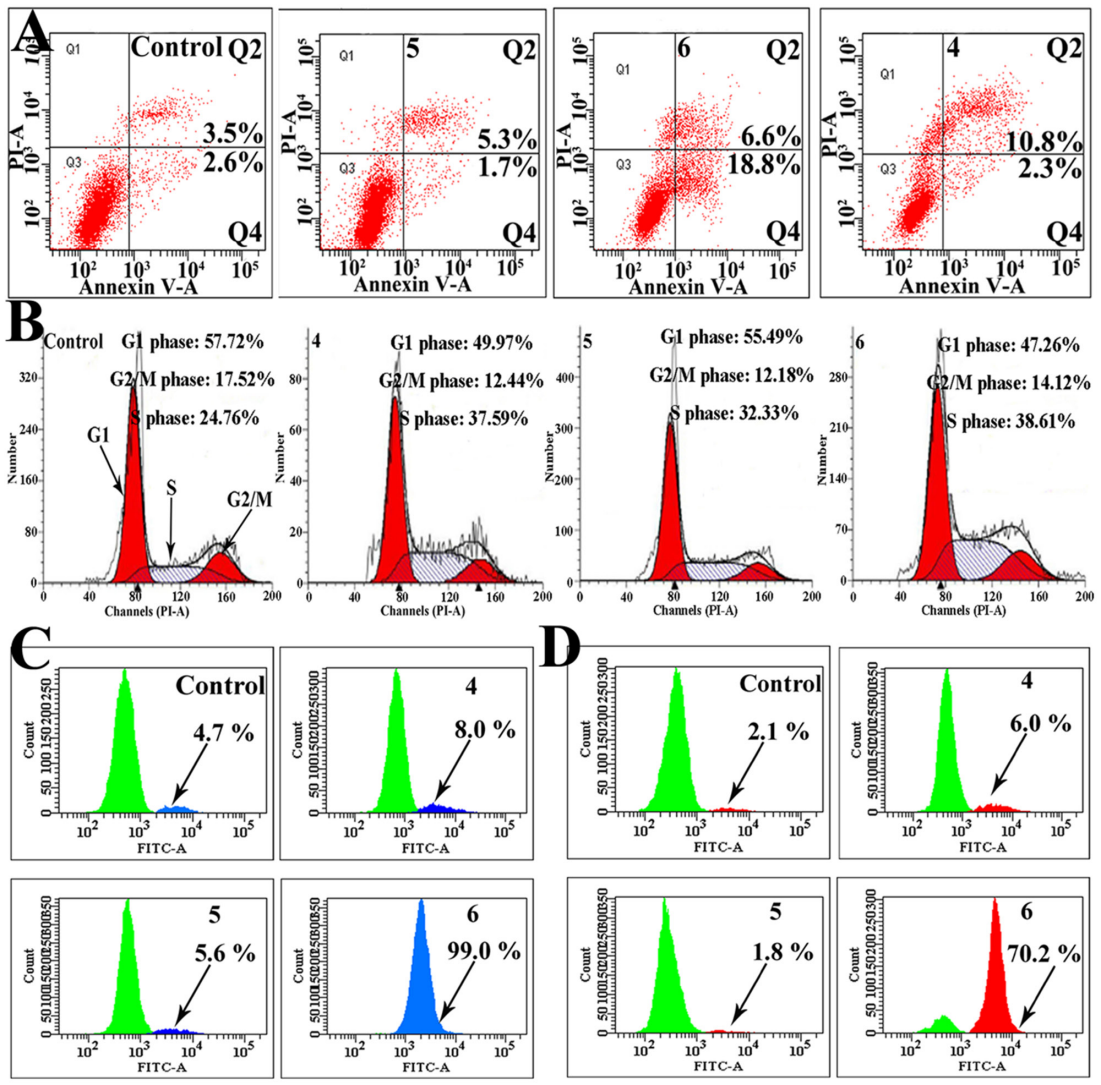
Complexes 4–6 induced apoptosis by triggering caspase-3/9 activity and caused S phase arrest in BEL-7404 cells **(A)** Effect of cell apoptosis of BEL-7404 treated with complexes 4–6 (10 *μ*M) for 24 h compared with the untreated cells. **(B)** Cell cycle effects of BEL-7404 cells treated with complexes 4–6 at 10 *μ*M for 48 h by flow cytometry. **(C)** and **(D)** The caspase-3 (C) and caspase-9 (D) protein expression was assessed by flow cytometry following treatment of BEL-7404 cells with complexes 4–6 (10 *μ*M) for 24 h.

Platinum(II)-based drugs were widely used in anticancer chemotherapies, therefore, we performed experiments to test the potency of chiral platinum(II) complexes 4–6 for inducing senescence and apoptosis in BEL-7404 cells. Figure 2D shows that BEL-7404 cells became aged morphologically 7 days after treatment with complexes 4–6. Treatment of BEL-7404 cells with complex 6 displayed a higher level of senescence (enlarged/flattened morphology, blue coloration) compared to the cells treated with complexes 4 and 5. We next investigated the effect of complexes 4–6 on the induction of apoptosis. BEL-7404 cells were incubated with complexes 4 (10 and 20 *μ*M), 5 (10 and 20 *μ*M) and 6 (5, 10 and 20 *μ*M) for 24 h, respectively, and then these cells staining with PI and Annexin-V-FITC were determined by flow cytometry (Figure 3A and Supplementary Figure 18). As shown in Figure 3A and Supplementary Figure 18, after treated of complexes 4–6 with 10 *μ*M for 24 h, the percentage of apoptotic cells (including early-stage and late-stage, Q2+Q4) was 13.1%, 7.0% and 25.4%, respectively, whereas the normal cells was only 6.1%. We further investigated the ability of complexes 4–6 (5, 10 and 20 *μ*M) to induce BEL-7404 cell cycle arrest (Figure 3B and Supplementary Figure 19). Cells were treated with complexes 4–6 at 10 *μ*M for 48 h, the cell cycle arrest was determined by using flow cytometry. As shown in Figure 3B and Supplementary Figure 19, the percentage of cells at the S phase is 24.76% in the untreated group (Figure 3B). However, treated with complexes 4–6 at 10 *μ*M increased the percentage of these cells at S phase to 37.59%, 32.33% and 38.61%, respectively. These results showed that complexes 4–6 caused S phase arrest in BEL-7404 cells. Moreover, the critical apoptotic events of cleavage of caspase-9 and caspase-3 were also induced (Figure 3C and 3D). Altogether, these results clearly show that complexes 4–6 could induce BEL-7404 cell senescence and apoptosis.

### Selective binding of chiral platinum(II) complexes to G4 DNA and modulation of telomeres conformation

**Figure 4:**
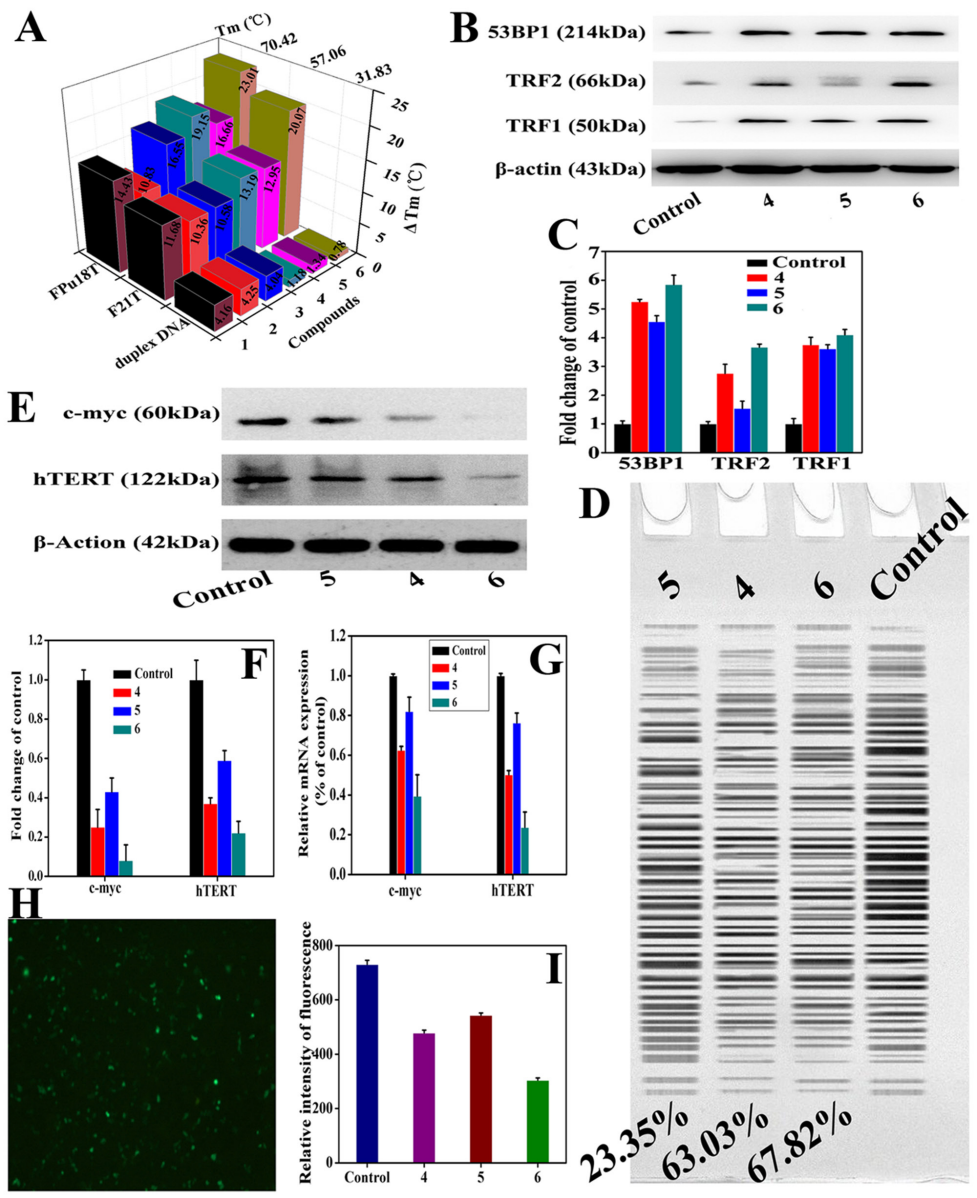
Complexes 4–6 induced telomeres damage and inhibited the telomerase activity through directly regulating the mRNA level of c-myc promoter (Pu27) **(A)** ΔTm data (°C) of 1.0 *μ*M HTG21, Pu39 and c-myc G4s and duplex DNA (F32T+H20M) treated with complexes 1–6 at 0–2.0 *μ*M were evaluated by RT-PCR. **(B)** The levels of TRF2, 53BP1, and TRF1 in BEL-7404 cells treated with complexes 4–6 at 10 *μ*M for 24 h were examined by Western blot. **(C)** The whole-cell extracts were prepared and analyzed by Western blot analysis using antibodies against TRF2, 53BP1, and TRF1. The same blots were stripped and re-probed with β-actin antibody to show equal protein loading. Western blotting bands from three independent measurements were quantified with Image J. in (B). **(D)** The influence of complexes 4–6 (10 *μ*M) on the telomerase activity of the BEL-7404 cells for 24 h. **(E)** The investigations of the expression of c-myc and hTERT in the BEL-7404 tumor cells when incubated with complexes 4–6 (10 *μ*M) for 24 h. C-myc and hTERT protein levels in BEL-7404 cells were analyzed by western blot. **(F)** The whole-cell extracts were prepared and analyzed by Western blot analysis using antibodies against c-myc and hTERT. The same blots were stripped and re-probed with β-actin antibody to show equal protein loading. Western blotting bands from three independent measurements were quantified with Image J. in (E). **(G)** qRT-PCR analysis of the expression levels of hTERT and c-myc in the BEL-7404 cells treated with complexes 4–6 (10 *μ*M). The BEL-7404 cells (5×10^5^) were treated with complexes 4–6 (10 *μ*M) for 24 h. The total RNA in the cells was extracted and subjected to reverse transcription, followed by PCR for c-myc, hTERT, and GAPDH (control). **(H)** and **(I)** The investigations of the role of the transfections of EGFP plasmid vector (H) and c-myc plasmid vector (I) in the BEL-7404 tumor cells when incubated with complexes 4–6 (10 *μ*M) for 24 h. First, 2.0 μg of EGFP-carrying plasmid vector or 2.0 μg of c-myc-carrying plasmid vector was cotransfected into BEL-7404 cells using Lipofectamine 2000 (Invitrogen, Grand Island, NY, USA). Complexes 4–6 (10 *μ*M) were then added, respectively, into medium at 6.0 h after transfection of c-myc plasmid. At another 24 h after treatment with complexes 4–6 (10 *μ*M), these cells were imaged using Nikon TE2000 (Japan) scanning fluorescent microscope or were examined by Multimodel Plate Reader with luciferase reporter gene assay kit.

Our previous data show that chiral 4-(2,3-dihydroxypropyl)-formamide oxoaporphine (FOA) has the ability to stabilize G-quadruplex DNA [68]. We asked whether chiral platinum(II) complexes 4–6 have the ability to bind to G-quadruplex DNA. To address this, FRET assays, CD spectroscopy, G4-FID assays, and fluorescence titration analysis were performed. FID assays showed that chiral platinum(II) complexes 4–6 selectively bind to Pu27 (c-myc) and HTG21 (human telomeric) G4s over other DNA (Supplementary Figure 20 and Supplementary Table 6), as well as higher affinity than that of the ligands 1–3. For G4-HTG21, the ^G4^DC_50_ values of complexes 4–6 were 1.23, 1.26 and 0.99 *μ*M, respectively; while, for G4-Pu27, the ^G4^DC_50_ values were 1.01, 0.89, and 0.82 *μ*M, respectively. In addition, the ^ctDNA^DC_50_/^G4^DC_50_ ratio of the HTG21 and Pu27 G4s treated with complexes 4–6 was in the range of 50.54-90.65 and 71.56-110.67 folds, respectively. To further evaluate the binding ability of complexes 4–6 to Pu27 and HTG21G4s, CD spectroscopy and fluorescence titration analysis were performed. The results of CD spectroscopy suggested that complexes 4–6 were able to induce Pu27 and HTG21 G4s to fold into a parallel conformation and mixed G4 structure in the absence/presence of K^+^ (Supplementary Figures 21–24 and Supplementary Table 7). The quenching ability of complexes 4–6 to HTG21 and Pu27 G4 DNA fluorescence can be quantitatively estimated by their respective quenching constant (Kq), which was derived from the Stern-Volmer quenching equation. The Kq values for complexes 4–6 were 4.60×10^4^, 4.36×10^4^ and 4.65×10^4^ (for HTG21 G4), and 1.02×10^5^, 8.80×10^4^ and 1.38×10^5^ (for Pu27 G4), respectively (Supplementary Table 8, Supplementary Figures 25 and 26), which indicated that complex 6 has a greater binding affinity to G4s DNA than complexes 4 and 5. In addition, the data from CD spectra and fluorescence titration analysis demonstrated that the binding of complexes 4–6 with G-quadruplex DNA might increase the stability of the structure of Pu27 and HTG21 G4-DNA (Supplementary Figures 21–26), suggesting that complexes 4–6 might interact with the loops and grooves of G-quadruplex, and thus interfering with DNA function, which is consistent with other groups’ reports [19, 68, 74]. Finally, the selective binding of complexes 4–6 towards G4 DNA was analyzed by FRET-melting assays. As shown in Figure 4A, Supplementary Table 9 and Supplementary Figures 27-30, the Δ*T*_m_ value of F21T was 13.19, 12.95, and 20.07 °C in the presence of complexes 4–6, respectively and the Δ*T*_m_ value of FPu18T was 19.15, 16.66, and 23.01 °C, respectively. In contrast, the Δ*T*_m_ value for the F32T+H20M duplex DNA in the presence of complexes 4–6 was 1.18, 1.34, and 0.78 °C under the same conditions (Figure 4A). Altogether, our data indicated complexes 4–6, especially complex 6, is the best binder to G4 DNA, which caused DNA complex displaying the highest thermodynamic stability.

As mentioned above, G4s structures widely exist in telomeres, we therefore monitored the effect of chiral platinum(II) complexes on function and activity of telomerase. To address this, we first determined the effect of complexes 4–6 on the expression of some telomeric DNA-associated proteins. As shown in Figure 4B and 4C, complexes 4–6 significantly induced the expression of TRF1, TRF2 and 53BP1 at protein level, indicating that complexes 4–6 could induce telomere dysfunction/damage (DNA damage). We next evaluated the activity of telomerase by TRAP assay. As shown in Figure 4D, the inhibitory ratio of telomerase activity induced by 10 *μ*M of complex 6 reached 67.82%, while that induced by complexes 4 and 5 only reached 63.03% and 23.35%, respectively, which demonstrated that complex 6 was the strongest telomerase inhibitor.

### Chiral platinum(II) complexes inhibit the telomerase activity through directly regulating c-myc/ hTERT promoter activity

To further investigate the mechanism(s) how chiral platinum(II) complexes regulate telomerase activity, two genes (hTERT and c-myc) critically associated with telomerase activation were investigated. BEL-7404 cells were treated with chiral platinum(II) complexes 4–6 at 10 *μ*M for 24 h, and then western blot and RT-PCR were performed to exam the expression of hTERT and c-myc at mRNA and protein levels. Interesting, we found that complex 6 could significantly attenuate the expression of these two genes in BEL-7404 cells compared to that of complexes 4 and 5 (Figure 4E–4G). We further generated a GFP reporter system linked with c-myc or hTRET promotor which bears G4 DNA sequence. The reporter plasmids were transfected into BEL-7404 cells, and then the cells were treated with 10 *μ*M chiral platinum(II) complexes for 24 h. We found that chiral platinum(II) complexes inhibited the fluorescence intensity of GFP reporter remarkably (Figure 4H), further confirmed the interaction between complexes 4–6 and G4s in c-myc or hTRET promoter (Figure 4I). Altogether, these results indicated that chiral platinum(II) complexes inhibited the telomerase activity, at least, *via* directly down-regulating c-myc/ hTRET gene in these cells.

### Acute toxicity studies

We further evaluated the safety of chiral platinum(II) complex 6 by using KM mice via the tail vein injection with a single dose of complex 6 at 15, 12, 9.6 and 7.8 mg/kg, respectively. After 14 days, the mortality rate of mice was calculated (Supplementary Figure 31 and Supplementary Table 10). We found that 7.8 mg/kg complex 6 did not kill any animal or cause any abnormality. However, at higher doses (>7.8 mg/kg), death of mice occurred. Thus, the doses of 7.8 mg/kg of complex 6 were employed in the further *in vivo* experiment.

We then assessed the *in vivo* toxicity of multiple doses of complex 6. The mice was treated with complex 6 at 7.8 mg/kg daily or per two day by iv injection up to 7 days, and then monitored for another week. Moderate body weight increase was observed in mice treated with complex 6, compared to the control, indicating that injection with complex 6 at 7.8 mg/kg daily was safe for mice.

### Chiral platinum(II) complex 6 inhibits tumor growth *in vivo*

**Figure 5:**
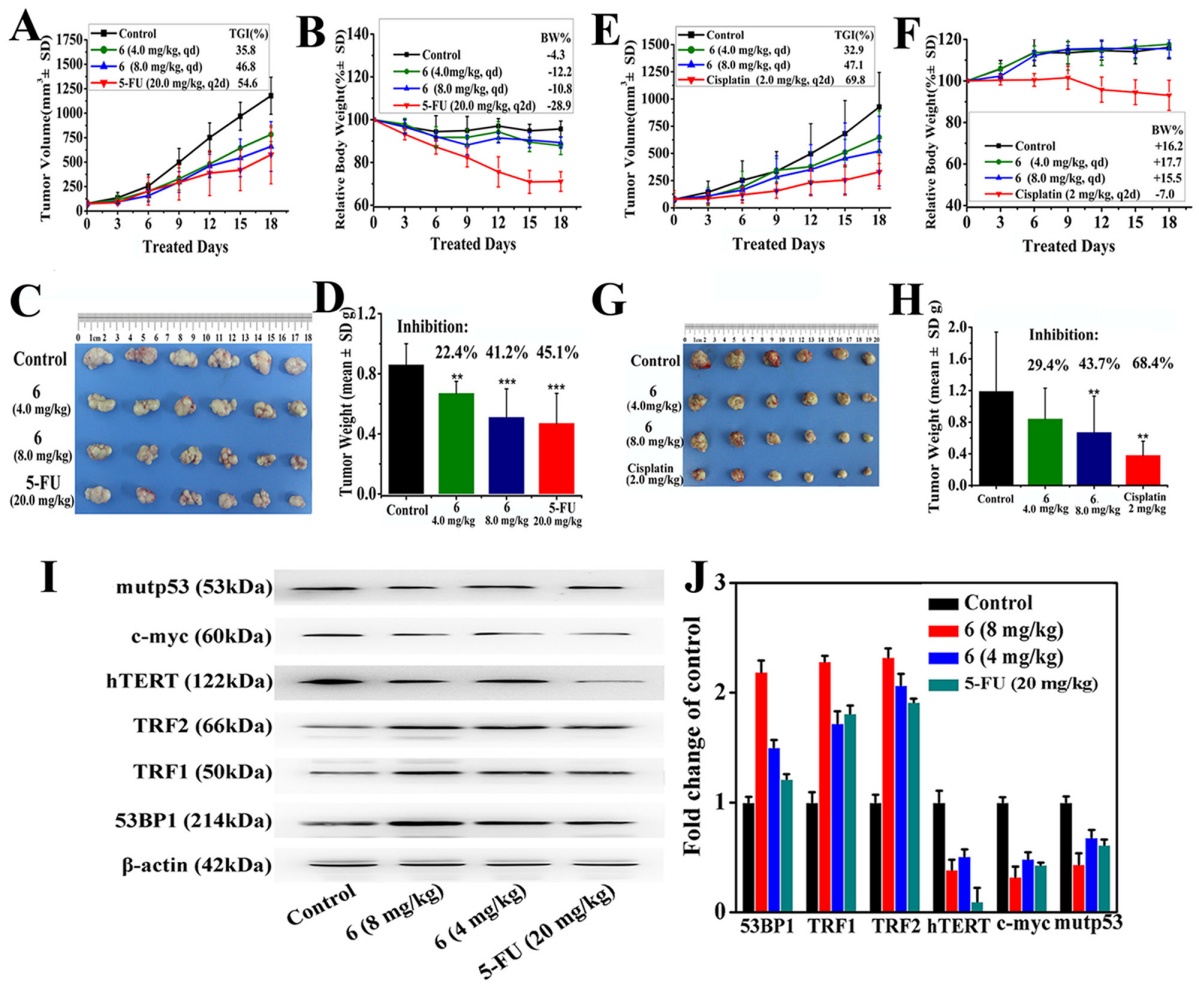
Complex 6 exhibited antitumor activity in BEL-7404 and BEL-7402 xenograft models **(A–D)** *In vivo* tumor growth inhibition activity of complex 6 (4.0, 8.0 mg/kg/d), and 5-FU (20 mg/kg/2 days) treated with BEL-7404 xenograft model. **(E–H)** *In vivo* tumor growth inhibition activity of complex 6 (4.0, 8.0 mg/kg/d) treated of BEL-7402 model with 4.0 and 8.0 mg/kg/d, comparing with cisplatin (2 mg/kg/2 days). (A) and (E). Changes in tumor volume between treatment groups (including complex 6 (4.0, 8.0 mg/kg/d), 5-FU (20 mg/kg/2 days) or cisplatin (2 mg/kg/2 days)) and vehicle (saline) group of BEL-7404 tumor-bearing mice and BEL-7402 tumor-bearing mice. Data of tumor growth were tracked by the mean tumor volume (mm^3^) ± SD (n = 6) and calculated as percent TGI (tumor growth inhibition, %) values. (B and F) Relative body weight change by considering the body weight at the start of the treated group as 100%, the percent weight loss or gain was calculated on subsequent days of treatment. (C) and (G) Tumor weight between treatment groups and vehicle (saline) group of BEL-7404 tumor-bearing mice and BEL-7402 tumor-bearing mice. (***) *P*<0.05, (**) *P*<0.05, *p vs* vehicle control. (D) and (H) Photographs of harvested tumors from vehicle group and each treatment groups. **(I)** The expression protein level of TRF1, mutp53, hTERT, TRF2, c-myc, and 53BP1 were analyzed by western blot in BEL-7404 xenograft models treated with 8 mg/kg and 4 mg/kg complex 6, and 20 mg/kg 5-FU, respectively. **(J)** The whole-BEL-7404 xenograft model extracts were prepared and analyzed by Western blot analysis using antibodies against TRF1, mutp53, hTERT, TRF2, c-myc, and 53BP1. The same blots were stripped and re-probed with β-actin antibody to show equal protein loading. Western blotting bands from three independent measurements were quantified with Image J. in (I).

The apoptotic action of chiral platinum(II) complex 6 via its binding to G4s and regulating telomerase activity prompted us to further investigate the anti-tumor activity of chiral platinum(II) complex 6 *in vivo* by examining growth repression of xenograft tumors in nude mice. *In vivo* anticancer efficacy of chiral platinum(II) complex 6 was evaluated using BEL-7404 and BEL-7402 xenograft mouse models. BEL-7404 and BEL-7402 cells were injected into nude mice. The mice were randomized into the control group, complex 6-treated group, and the positive control group (n = 6/group). When the volume of tumor was about 80 mm^3^, complex 6 was given at a high (8 mg/kg) and low (4 mg/kg) dosage daily for 18 days, 5-FU (20 mg/kg every two days) and Cisplatin (2 mg/kg every two days) were used as a positive control in the BEL-7404 and BEL-7402 xenograft model, respectively. As shown in Figure 5, tumor growth was significantly reduced with chiral platinum(II) complex 6 treatment both in BEL-7404 (46.8% inhibition in weight, 8 mg/kg; 35.8% inhibition in weight, 4 mg/kg; p<0.05) and BEL-7402 (47.1% inhibition in weight, 8 mg/kg; 32.9% inhibition in weight, 4 mg/kg; p<0.05) xenograft models. These data indicated that complex 6 presented a dose-dependent tumor growth inhibition to BEL-7404 and BEL-7402 xenograft tumors. 5-FU and Cisplatin showed higher inhibitory activity to BEL-7404 and BEL-7402 xenograft model (Figure 5A–5H and Supplementary Tables 11–16), as compared with mice injected with complex 6, however, serious body weight loss was observed (Figure 5B and 5F). On the contrary, the nude mice did not display any discomfort or discernible side effects in diet intake or overall body weight during administration of complex 6 (Figure 5B and 5F).

**Figure 6:**
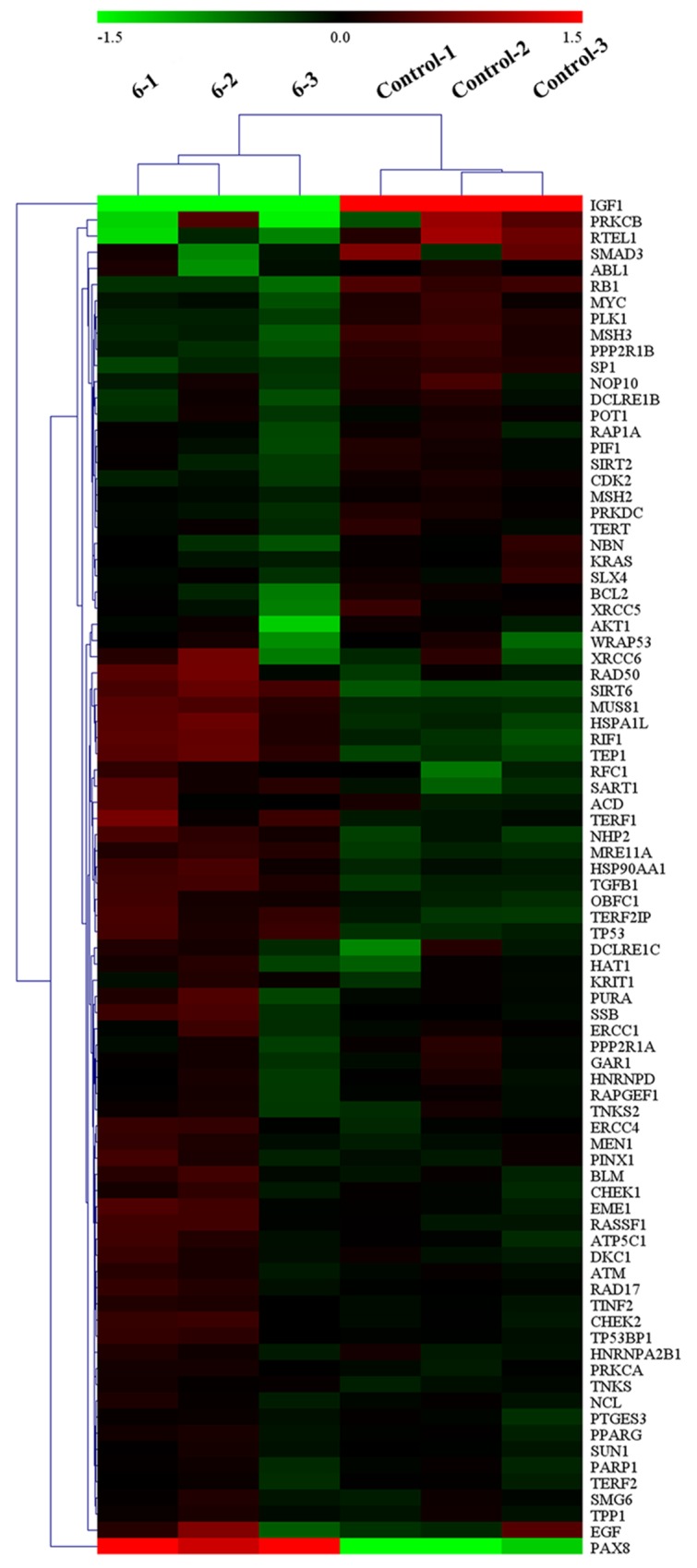
RT-qPCR array for determining mRNA levels of telomeres/telomerase-related gene expressions in BEL-7404 cells after treatment with complex 6 at 10 *μ*M for 24 h

To determine the mechanism(s), we further explored the effect of complex 6 on the expression of telomeres/telomerase-related genes (such as TRF1, hTERT, TRF2, c-myc, and 53BP1) in the BEL-7404 model. Firstly, RT-qPCR array was used to determine mRNA levels of telomeres/telomerase-related gene expressions in BEL-7404 cells when the cells were treated with complex 6. We found that some genes related with telomerase activity were changed (Figure 6 and Supplementary Table 17), as expected. Secondly, these telomerase-related genes were determined by western blot. As shown in Figure 5I and 5J, like 5-Fu, complex 6 significantly up-regulated the protein expression of TRF1, TRF2, and 53BP1, however, down-regulated the expression of mutp53, c-myc, and hTERT, when the BEL-7404 xenograft mice were treated with complex 6. These results were consistent with RT-qPCR array assay.

## DISCUSSION

Several research groups have reported that DNA forms a G-quadruplex structure, which is widely present in human telomeric DNA, transcription start sites, and the promoter regions of genes, such as bcl-2, c-myc, k-ras, and c-kit, suggesting that G-quadruplex structures may play a critical role in the control of a variety of cellular processes, including telomere maintenance, gene replication, transcription and translation [75–77]. More interestingly, the association of the special structure of G-quadruplex with human diseases has been reported, which makes the G-quadruplex a potent therapeutic target. Therefore, a variety of small molecules have been identified and designed in an effort to target the G-quadruplex.

Here, three chiral platinum(II) complexes 4–6 with different chiral FOA ligands were synthesized and fully characterized (Figure 1). We found that complexes 4–6 displayed much higher antitumor activities compared to their corresponding chiral ligands *in vitro* and *in vivo* (Figures 2 and 5). The chiral platinum(II) complexes could induce BEL-7404 cell senescence and apoptosis (Figure 3). Interestingly, the cells arrested in S phase were observed after chiral platinum(II) complexes treatment. Importantly, the antitumor activity of chiral platinum(II) complexes also has been observed in the BEL-7404 and BEL-7402 xenograft mouse models (Figure 5). In this regard, our findings provided compelling evidence, demonstrating that chiral platinum(II) complexes might be a novel anticancer drug candidate, however, the knowledge about their mechanism of action is limited.

Our previous data showed that chiral 4-(2,3-dihydroxypropyl)-formamide oxoaporphine (FOA) has the ability to stabilize G-quadruplex DNA [68], which prompted us to ask whether chiral platinum(II) complexes 4–6 have the ability to bind G-quadruplex DNA. Indeed, using various biochemical and spectroscopic assays, we showed that chiral platinum(II) complexes 4–6 directly targeted telomeric G4s sequence, as well as c-myc/hTERT G4s in BEL-7404 cells, and thus resulted in the inhibition of telomerase activity, especially complex 6 treatmented group. The significantly improved biological behavior by complex 6 may be correlated with S-(–)-FOA ligand, similar to previous reported results of [PtCl_2_(S-(–)-BINAP)_2_] and [PtCl_2_(S-(–)-DABN)_2_] [61, 62]. In addition, c-MYC expression is physiologically induced during G1/S-phase progression and deregulated in a variety of malignant tumors. It is well known that G-quadruplex is widely present in the MYC promoter region, and we found that stabilization of the G-quadruplex by interacting with chiral platinum(II) complexes 4–6 led to inhibition of MYC expression (Figure 4E–4G). We also observed that TERT, which codes for the catalytic subunit of telomerase and is regulated by MYC, was significantly down-regulated by chiral platinum(II) complexes, which further indicated the effect of chiral platinum(II) complexes on telomerase activity.

We evaluated the *in vivo* activity of chiral platinum(II) complexes in BEL-7404 and BEL-7402 xenograft mouse models. We demonstrated that complex 6 presented significant growth inhibition to BEL-7404 and BEL-7402 xenograft tumors. We further explored the effect of complex 6 on the expression of telomeres/telomerase-related genes (such as TRF1, mutp53, hTERT, TRF2, c-myc, and 53BP1) in the BEL-7404 model. Our data indicated that treatment with complex 6 achieved significant downregulation of telomerase activity in xenograft models *in vivo*, which was associated with antitumor activity. Although, 5-FU and Cisplatin showed higher tumor inhibitory activity to xenograft model (Figure 5A–5H), serious body weight loss was observed as compared with mice injected with complex 6.

**Figure 7:**
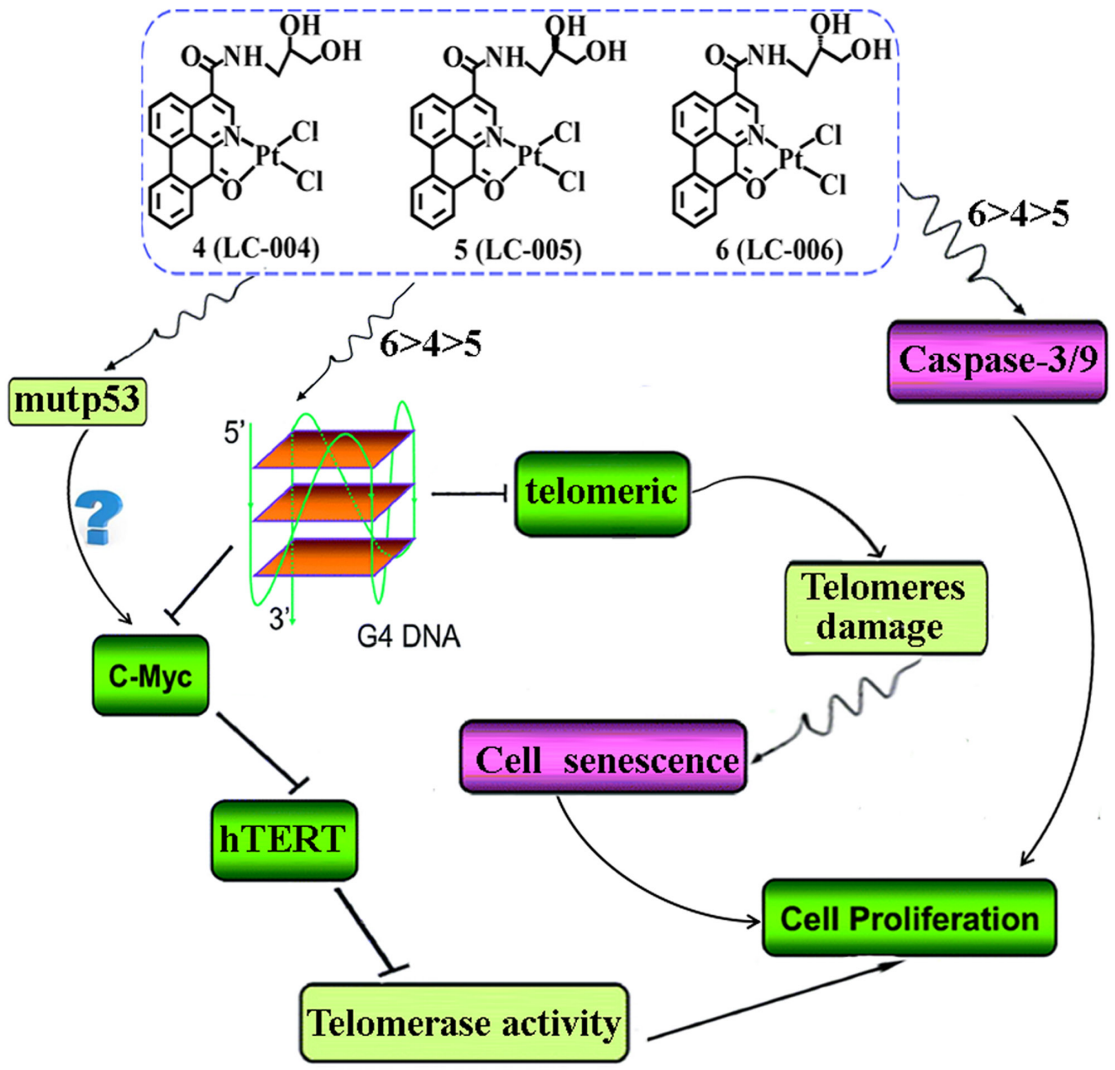
Proposed antitumor mechanisms for chiral platinum(II) complexes 4–6

In summary, *in vitro* and *in vivo* studies showed that chiral platinum(II) complexes had a significant inhibitory effect on both tumor volume and weight in mouse model. The chiral platinum(II) complexes caused effective reductions in telomerase activity that led to induction of antitumor activity, at least, via directly down-regulating c-myc/hTRET gene in these cells (Figure 7). Although 5-FU and cisplatin displayed higher tumor growth inhibition, their toxicity was apparent, as evident from the pronounced loss of body weight. Therefore, our results clearly demonstrated that chiral platinum(II) complexes (especially complex 6) are relatively safe antitumor agents and can inhibit the growth of BEL-7404 and BEL-7402 tumors *in vivo*.

## MATERIALS AND METHODS

### Synthesis of 4-(2,3-dihydroxy propyl)-formamide oxoaporphine alkaloids

Synthesis and characterization of the chiral ligands 1–3 have been reported in our previous work [68]. Preparation of dimethyl 7-oxo-7*H*-dibenzo[de,g]quinoline-4,5-dicarboxylate (I) was carried out by Tang and Chen reported method [68, 78]. Hydrolysis of (I) using potassium hydroxide gave 4,5-dicarboxyl-7-oxo-7*H*-dibenzo[de,g]quinoline (II) in 92.0% yield. 4-carboxyl-7-oxo-7*H*-dibenzo[de,g]quinoline (III) was obtained in 73.0% yield by decarboxylation of II in diphenyl ether at 190 °C for 1.5 h. Cyclization of III, PyBOP and R/S-(±)-3-amino-1,2-propanediol, R-(+)-3-amino-1,2-propanediol or S-(−)-3-amino-1,2-propanediol in N,N-dimethyl acetamide (DMA) at 65.0 °C for 1.0 h afforded the chiral FOA ligands R/S-(±)-FOA (1), R-(+)-FOA (2) and S-(–)-FOA (3) in 61.8-78.6% yield.

### Synthesis of complex 4

The Pt(II) complex 4 (bottle-green block product) was prepared by treating 0.05 mmol *cis-*Pt(DMSO)_2_Cl_2_ with 0.05 mmol ligand 1 in CH_3_OH/H_2_O (3:1) at 80 °C for 8 h. Yield (0.0253 g, 82.40%).^1^H NMR (500 MHz, DMSO-*d*_*6*_) δ 9.06 (s, 1H), 8.94-8.92 (m, 1H), 8.81 (d, *J* = 7.1 Hz, 1H), 8.60 (d, *J* = 8.1 Hz, 1H), 8.38–8.35 (m, 1H), 8.32 (dd, *J* = 7.9, 1.4 Hz, 1H), 8.04 (dd, *J* = 8.5, 7.4 Hz, 1H), 7.93–7.88 (m, 1H), 7.71–7.66 (m, 1H), 4.95 (d, *J* = 4.1 Hz, 1H), 4.68 (s, 1H), 4.37 (s, 1H), 3.75 (s, 1H), 3.60–3.53 (m, 1H). ^13^C NMR (125 MHz, DMSO-*d*_*6*_) δ 181.7, 166.5, 147.0, 143.6, 135.3, 135.1, 134.1, 132.5, 131.3, 130.0, 128.4, 128.4, 127.3, 126.4, 124.7, 124.2, 70.7, 64.5, 43.6. ESI-MS m/z: 657.1 [M–Cl+DMSO]^+^. IR (KBr, cm^-1^): 3269, 3071, 2930, 2720, 2368, 1742, 1641, 1602, 1564, 1506, 1473, 1388, 1353, 1317, 1283, 1262, 1218, 1199, 1163, 1105, 1039, 968, 938, 875, 831, 770, 694, 604, 573. Elemental analysis calcd. (%) for C_21_H_19_Cl_2_N_2_O_4_Pt: C 40.08, H 3.04, N 4.45; found: C 40.12, H 3.05, N 4.43.

### Synthesis of complex 5

The Pt(II) complex 5 (bottle-green block product) was prepared by treating 0.05 mmol *cis-*Pt(DMSO)_2_Cl_2_ with 0.05 mmol ligand 2 in CH_3_OH/H_2_O (3:1) at 80 °C for 8 h. Yield (0.022 g, 72.10%). ^1^H NMR (500 MHz, DMSO-*d*_*6*_) δ 9.06 (s, 1H), 8.93–8.91 (m, 1H), 8.81 (d, *J* = 7.4 Hz, 1H), 8.61 (d, *J* = 8.1 Hz, 1H), 8.38 (d, *J* = 8.5 Hz, 1H), 8.33 (dd, *J* = 7.8, 1.1 Hz, 1H), 8.08–8.01 (m, 1H), 7.94–7.88 (m, 1H), 7.70–7.67 (m, 1H), 4.95 (d, *J* = 5.0 Hz, 1H), 4.67 (t, *J* = 5.3 Hz, 1H), 4.37 (d, *J* = 4.0 Hz, 1H), 3.77-3.75 (m, 1H), 3.58-3.54 (m, 1H). ^13^C NMR (125 MHz, DMSO-*d*_*6*_) δ 181.7, 166.5, 147.0, 143.7, 135.3, 135.1, 134.1, 132.5, 131.3, 130.0, 128.5, 128.4, 127.3, 126.5, 124.8, 124.2, 70.8, 64.5, 43.6. ESI-MS m/z: 719.2 [M–Cl+DMSO+2CH_3_OH]^+^. IR (KBr, cm^-1^): 3406, 3066, 3000, 2912, 2346, 1599, 1564, 1509, 1457, 1407, 1317, 1286, 1202, 1118, 1026, 944,764, 691, 606, 521 cm^−1^. Elemental analysis calcd (%) for C_21_H_19_Cl_2_N_2_O_4_Pt: C 40.08, H 3.04, N 4.45; found: C 40.05, H 3.06, N 4.41.

### Synthesis of complex 6

The Pt(II) complex 6 (bottle-green block product) was prepared by treating 0.05 mmol *cis-*Pt(DMSO)_2_Cl_2_ with 0.05 mmol ligand 3 in CH_3_OH/H_2_O (3:1) at 80 °C for 8 h. Bottle-green products for analysis were harvested. Yield (0.0272 g, 88.50%). ^1^H NMR (500 MHz, DMSO-*d*_*6*_) δ 9.06 (s, 1H), 8.92–8.90 (m, 1H), 8.80 (d, *J* = 7.4 Hz, 1H), 8.60 (d, *J* = 8.1 Hz, 1H), 8.37 (d, *J* = 8.5 Hz, 1H), 8.32 (dd, *J* = 7.8, 1.1 Hz, 1H), 8.07–8.01 (m, 1H), 7.93–7.87 (m, 1H), 7.70–7.67 (m, 1H), 4.94 (d, *J* = 5.0 Hz, 1H), 4.67 (t, *J* = 5.3 Hz, 1H), 4.36 (s, 1H), 3.77–3.74 (m, 1H), 3.59–3.53 (m, 1H). ^13^C NMR (125 MHz, DMSO-*d*_*6*_) δ 181.7, 166.5, 147.0, 143.6, 135.3, 135.1, 134.1, 132.5, 131.3, 130.0, 128.4, 128.4, 127.3, 126.4, 124.7, 124.2, 70.7, 64.5, 43.6. ESI-MS m/z: 657.1 [M–Cl+DMSO]^+^. IR (KBr, cm^−1^): 3481, 3736, 3066, 2863, 2363, 1997, 1638, 1602, 1542, 1473, 1457, 1410, 1388, 1353, 1317, 1281, 1262, 1218, 1199, 1163, 1106, 1095, 1040, 966, 938, 916, 875, 831, 798, 765, 694, 604, 573, 494. Elemental analysis calcd. (%) for C_21_H_19_Cl_2_N_2_O_4_Pt: C 40.08, H 3.04, N 4.45; found: C 40.07, H 3.09, N 4.47.

### Materials, methods and cell lines

All the materials, instrumentation, and the detailed procedures for other experimental methods are described in supporting information. The antitumor mechanisms of complexes 4–6 have been reported by Chen and co-workers [51, 68, 79]. The TRAP-silver staining assay of complexes 4–6 were performed as reported by Mikami-Terao, Chao and Chen reported [51, 80, 81]. In addition, BEL-7404 and BEL-7402 xenograft mouse models were purchased from Beijing HFK Bioscience Co., Ltd (Beijing, China, approval No. SCXK 2014-004). The animal procedures were approved by the Institute of Radiation Medicine Chinese Academy of Medical Sciences (Tian Jin, China, approval No. SYXK 2014-0002). And all of the experimental procedures were carried out in accordance with the NIH Guidelines for the Care and Use of Laboratory Animals. Animal experiments were approved by the Animal Care and Use Committee of the Institute of Radiation Medicine Chinese Academy of Medical Sciences. Moreover, abbreviations, the human cell lines and DNA oligomers of used in this work are listed in Supplementary Table 1 and DNA oligomers were obtained from Shanghai Sangon Biological Engineering Technology & Services (Shanghai, China).

### *In vivo* tumor growth inhibition

To evaluate the vivo anticancer efficacy of complex 6, BEL-7404 and BEL-7402 xenograft mouse models (BALB/c nude mice, female for BEL-7404 model, 17–20 g, 6–7 weeks old; male for BEL-7402 model, 18-21 g, 6–7 weeks old) were used. Furthermore, animal used, maximum tolerated dose (MTD) analysis, acute toxicity studies and antitumor activity toward BEL-7404 and BEL-7402 models *in vivo* of complex 6, was similar to Chen reported [68, 79].

### Abbreviations

The detailed for abbreviations, the human cell lines and DNA oligomers of used in this work are listed in Supplementary Table 1.

## SUPPLEMENTARY MATERIALS FIGURE AND TABLES






