# Supplementary material for: Chiral platinum (II)-4-(2,3-dihydroxypropyl)- formamide oxo-aporphine (FOA) complexes promote tumor cells apoptosis by directly targeting G-quadruplex DNA in vitro and in vivo
Source: Oncotarget. 2017 Jun 28;8(37):61982–97. doi: 10.18632/oncotarget.18778 (PMC5617480; doi:10.18632/oncotarget.18778)
Supplement: Supplementary file 1 [file oncotarget-08-61982-s001.pdf]

Chiral platinum(II)-4-(2,3-dihydroxypropyl)-formamide oxoaporphine (FOA) complexes promote tumor cells apoptosis by directly targeting G-quadruplex DNA *in vitro* and *in vivo*

SUPPLEMENTARY MATERIALS

Materials

Tris, RNase A, and propidium iodide (PI) were purchased from Sigma. The antibody of mutp53, 53BP1, TRF1, TRF2, hTERT, c-myc, and caspase-3/9 were purchased from Abcam. Unless otherwise stated, spectroscopic titration experiments were carried out in TBS (10 mM pH7.35 Tris-HCl, containing 100 mM KCl). The total RNA isolation kit and the two-step RT-PCR kit were purchased from TIANGEN. DNA oligomers (highly polymerized stored at 4 °C; long-term storage at −20 °C) are listed in Supplementary Table 1 and were obtained from Shanghai Sangon Biological Engineering Technology & Services (Shanghai, China). All tumor cell lines (HeLa, BEL-7402, MGC80-3, BEL-7404, A549, Hep-G2 cancer-cell lines and normal liver cell line HL-7702) were obtained from the Shanghai Institute for Biological Science (China). Stock solutions of all the compounds (2.0 mM) were made in DMSO, and further dilutions to working concentrations were made with corresponding buffer.

Instrumentation

Infrared spectra were obtained on a Perkine Elmer FT-IR Spectrometer. Elemental analyses (C, H, N) were carried out on a Perkin Elmer Series II CHNS/O 2400 elemental analyser. NMR spectra were recorded on a Bruker AV-500 NMR spectrometer. Fluorescence measurements were performed on a Shimadzu RF-5301/PC spectro fluorophotometer. ESI-MS (electrospray ionization mass spectrum) were recorded on a Bruker HCT Electrospray Ionization Mass Spectrometer (ThermalElctronic, USA). HPLC spectra were performed on Waters e2695 Spectrometer. The circular dichroic spectra of DNA were obtained on a JASCO J-810 automatic recording spectropolarimeter operating at 25 °C. The region between 200 and 400 nm was scanned for each sample. MTT assay was performed on M1000 microplate reader (Tecan Trading Co. Ltd, Shanghai, China). Cell cycle and apoptosis analysis was recorded on FACS Aria II Flow Cytometer (BD Biocsiences, San Jose, USA).

Cytotoxicity assay (MTT assay)

The cell culture was maintained on RPMI-1640 medium supplemented with 10% fetal bovine serum, 100 U/mL penicillin and 100 μg/mL streptomycin in 25 cm^2 culture flasks at 37 °C humidified atmosphere with 5% CO_2. All cells to be tested in the following assays have a passage number of 3-6.

The cells 5.0×10^3 (HeLa, BEL-7402, MGC80-3, BEL-7404, A549, and Hep-G2 cancer-cell lines and HL-7702 normal cells) per well were seeded in triplicate in 96-well plates and incubated for 24 h at 37 °C and 5.0% CO_2/95.0% air. Then graded amounts of compound were added to the wells in 10.0 μL of FBS free culture medium and the plates were incubated in a 5% CO_2 humidified atmosphere for 24 h and 48 h. Six replica wells were used as controls. Each the cells were grew for 12.0 h before treatment to reach 70.0% confluency and 20.0 μL of tested various concentrations of compounds were added to each well. The final concentration of the tested complexes or compounds were kept at 1.25, 2.5, 5, 10, 20 μM, respectively. After 24 h or 48 h of culture, 0.1 mg of MTT (in 20 μL of PBS) was added to each well, and cells were incubated at 37 °C for 6 h. The formed formazan crystals were then dissolved in 100 μL of DMSO and the absorbance was read by enzyme labeling instrument with 490/630 nm double wavelength measurement. The final IC_{50} values were calculated by the Bliss method (n = 5). All tests were repeated in at least three independent trials.

Uptake of Pt in BEL-7404 cells

The BEL-7404 cells (~10 million cells) were treated with complexes 4–6 (10 μM) for 8.0 h at 37.0 °C in a humidified 5.0% CO_2 incubator, comparing with cisplatin (10 μM). The spent media was removed, and the BEL-7404 cells were washed with 5.0 mL of PBS for three times, scraped, and collected in 5.0 mL of PBS. The scrapped cells were spun down, by centrifuging at 2500 rpm for 10.0 min. The cell pellet obtained was dissolved in 1.0 M NaOH (1.0 mL) and diluted with 2.0% (v/v) HNO_3 (5.0 mL) for determining whole cell Pt content. Another set was treated similarly, nuclear proteins, nuclear fraction, cytoplasmic protein, membrane proteins and other fraction were isolated as described by Chen and Schreiber et al [1–3]. And the each final solution was made up to 5.0 mL using 2.0% (v/v) HNO_3. The amount of Pt taken up by the cells was determined by ICP-MS. The instrument was calibrated for Pt using standard solutions containing 10, 50, 100, 500 and 1000 ppb Pt (platinum).

Apoptosis analysis

Apoptosis was detected by flow cytometric analysis of annexin V staining. Annexin V-FITC vs PI assay was performed as previously described [4, 5]. Briefly, adherent NEL-7404 cells were harvested and suspended in the annexin-binding buffer (5×10^5 cells/mL). Then, cells were incubated with annexin V-FITC and PI for 1.0 h at room temperature in the dark and immediately analyzed by flow cytometry. The data are presented as biparametric dot plots showing PI red fluorescence vs annexin V-FITC green fluorescence.

Cell cycle analysis

In cell cycle analysis, BEL-7404 cells cultured with complexes 4–6 were harvested, washed with phosphate-buffered saline (PBS, 1.0 mL), fixed with 70% ethanol, and these BEL-7404 cells 1.0×10^6 were centrifuged and resuspended in a staining solution (0.5 mL of PBS containing 50.0 μg/mL PI and 75.0 kU/mL RNase A) for 30.0 min at room temperature in the dark. Finally, the BEL-7404 cell cycle was determined with FACS Calibur flow cytometer (BD) and analyzed using Cell Quest (BDIS) and ModFit LT (Verity Software House, Topsham, ME).

Determination of caspase-3/9 activity by flow cytometric analysis

Colorimetric analysis of caspase-9 and caspase-3 activation in BEL-7404 cells exposed to 10 μM of complexes 4–6 for 24.0 h were carried out using the CaspGLOW™ Fluorescein Active Caspase-9/Caspase-3 Staining Kit. The BEL-7404 cells treated with complexes 4–6 and the controlled BEL-7404 cells were harvested from RPMI 1640 medium with 10% FBS, in a density of 1.0×10^6 cells per milliliter. These cells were washed with PBS and centrifuged for 3 times, and were then mixed with 500 μL culture. 1.0 μL of FITC-LEHD-FMK or/and FITC-DEVD-FMK were consequently added and incubated for 2.0 h at 37 °C with 5% CO_2. The BEL-7404 cells were then determined with a FACS AriaII flow cytometer equipped with a 488 nm argon laser. Results were represented as the percent change on the activity comparing with the control group.

Western blot assay

The BEL-7404 Cells were treated with the same as the above for 24.0 h with complexes 4–6 (10 μM), and total proteins were extracted by lysis buffer RIPA. Then use BCA protein assay to measure concentrations of each sample. After electrophoresis on 10% PAGE-SDS gel and blotting, anti-mutp53, 53BP1, TRF1, TRF2, hTERT, c-myc antibody, or anti-β-actin primary antibody and the corresponding secondary antibody were used to detect target protein *via* standard washing and incubating steps.

Senescence induction and selective binding to G4s by 4–6 in BEL-7404 cells

Staining for SA-β-Gal activity was carried out according to Moore and Chen reports [6, 7]. In brief, After the long-term incubation with complexes 4–6, the growth medium was aspirated and the BEL-7404 cells were fixed in 2% formaldehyde/0.2% glutaraldehyde for 15.0 min at room temperature. The fixing solution was removed, and the cells were gently washed twice with PBS, and then stained using the β-Gal staining solution (containing 1.0 mg/mL of 5-bromo-4-chloro-3-indolyl-β-D-galactoside), and followed with incubation overnight at 37.0°C. The staining solution was removed, and the BEL-7404 cells were washed 3-5 times with PBS. The BEL-7404 cells were viewed under an optical microscope and photographed (Nikon Te2000, Japan). In addition, RNA extraction, RT-PCR, and the selective binding to G4s by complexes 4–6 was according to the our previous reported [1, 6, 9].

Gene expression by a panel of genes for RT-qPCR array

Differential expression of telomeres/telomerase-related genes was analyzed using the Telomeres & Telomerase PCR Array (RT^2 Profiler™ PCR Array Human Telomeres & Telomerase, PAHS-010Z, SABioscience, USA) obtained from Kangchen Bio-tech, Shanghai, China. RNA was extracted according to standard protocols and converted to first strand cDNA using the RT2 First Strand Kit. The template was added to an instrument specific, ready-to-use RT2 SYBR Green qPCR Master Mix. The resulting mixture was added to the wells (25.0 μL/well) of the PCR Array plate containing the telomere maintenance, telomere-associated complexes, telomere regulation and other genes associated with telomeres gene-specific primer sets (25 μL for 96-well plates) and PCR was performed. The threshold cycle (Ct) values for all the genes on each PCR Array were calculated using the instrument specific software and the fold-changes in gene expression for pair-wise comparison were calculated using the $2^{-\Delta Ct}$ method.

TRAP Assay (TRAP-silver staining assay)

Each telomerase extract was prepared from BEL-7404 cells. A modified version of the TRAP assay was used [8–11]. The PCR was performed in a final 50.0 mL reaction volume composed of reaction mix (45.0 mL) containing pH 8.0 Tris-HCl (20.0 mM), deoxynucleotide triphosphates (50.0 mM), BSA (20 mg/mL), primer

H21T (3.5 pmol; 5'-$G_3[T_2AG_3]_3$-3'), primer TS (18 pmol; 5'-AATCCGTCGAGCAGAGTT-3'), KCl (63 mM), EGTA (1 mM), $MgCl_2$ (1.5 mM), Tween-20 (0.005%), primer NT (7.5 pmol; 5'-ATCGCTTCTCGGCCTTTT-3'), TSNT internal control (0.01 amol; 5'-ATTCCGTCGAG CAGAGTTAAAAGGCCGAGAAGCGAT-3'), primer Cxext (22.5 pmol; 5'-GTGCCCTTACCCTTACCCT TACCCTAA-3'), Taq DNA polymerase (2.5 U), and telomerase (100 ng). Complexes or/and distilled water was added (5.0 mL). PCR was performed in an Eppendorf Master cycler equipped with a hot lid and incubated for 30.0 min at 30 °C, followed by 92 °C 30 s, 52 °C 30 s, and 72°C 30 s for 30.0 cycles. After amplification, 8.0 mL loading buffer (5×TBE buffer, 0.2% xylene cyanol, and 0.2% bromophenol blue) were added to the reaction. An 15.0 mL of aliquot was loaded onto a nondenaturing acrylamide gel (19:1, 16%) in 1×TBE buffer and resolved at 200.0 V for 1.0 h. Gels were fixed and then stained with $AgNO_3$ [8–11].

Transfection

In order to verify whether complexes 4–6 can directly regulate c-myc promoter (Pu27) and the expression of hTRET in BEL-7404 cells, consequently inhibiting the telomerase function, we constructed EGFP and c-myc gene vectors according to those illustrated in Chalfie, Chen and He previous work. The detailed procedures for the construct of gene (c-myc and EGFP genes) vectors, and the method of transfection assays have been reported by Chalfie, Chen and He [1, 6, 9, 12, 13]. In brief, each 8.0×10^5 BEL-7404 cells were grown in 3 cm Petri dishes, and after 24.0 h, DNA transfections were performed as follows. First, 2.0 µg of EGFP plasmid [12] or 2.0 µg c-myc plasmid [13] were co-tranfected into BEL-7404 cells using Lipofectamine 2000 (Invitrogen), respectively. Then, complexes 4–6 (10 μM) were added into medium after 6.0 h of transfection, respectively. After another 24.0 h of complexes 4–6 (10 μM) treatment, the BEL-7404 cells were imaged using Nikon TE2000 (Japan) scanning fluorescent microscope, were determined by Western blotting or studied by luciferase reporter gene assay kit.

In Vivo tumor growth inhibition

BALB/c nude mice (female for BBEL-7404 model, 17–20 g, 6–7 weeks old; male for BEL-7402 model, 18–21g, 6–7 weeks old) were housed at individual ventilated caging system (IVC Rack) with a sterile environment with conditions of constant photoperiod (12.0 h light/12 h dark at 25~28°C and 45~65% humidity). Nude mice inoculated subcutaneously with 5×10^7 BEL-7402 or BEL-7404 cells in right flank. When the tumor growth to the volume about 1500 mm^3, the mice were killed and the tumor tissue were cut into about 1.5 mm^3 small pieces, and then transplanted into the right flank of nude mice. When the average tumors reach a volume of about 80 mm^3, the mice were randomized into 4 groups (n=6), received the following treatments: (a) control, received saline solvent; (b) complex 6 at doses of 8.0 mg/kg daily (dissolve in saline, iv); (c) complex 6 at doses of 4.0 mg/kg daily; (d) in BEL-7404 model, 5-fluorouracil (5-Fu) as positive control at 20 mg/kg per two days (dissolve in saline, iv); in BEL-7402 model, cisplatin as positive control at 2 mg/kg per two days (dissolve in saline, iv).

The tumor volumes and body weight was monitored every 3 days, The tumor volumes were estimated by measuring the two dimensions of the tumors using caliper and calculated by the formula 1 [1, 6, 14]:

$$V = (\text{length} \times \text{width}^2)/2 \qquad (1)$$

On day 18, the mice were sacrificed, and tumor tissue were dissected out and weighted, tumor growth inhibition rate calculated using formulas 2 and 3 [1, 6, 15, 16]:

$$TGI\ (\%) = (\Delta V_c - \Delta V_t)/\Delta V_c \times 100\% \qquad (2)$$

$$IR(\%) = (W_c - W_t)/W_c \times 100\% \qquad (3)$$

Where ΔV_t = tumor volume$_{final}$ −tumor volume$_{initial}$ for compound treated group, ΔV_c=tumor volume$_{final}$ − tumor volume$_{initial}$ for vehicle control group; W_t and W_c mean the average tumor weight of complex-treated and vehicle controlled group respectively.

REFERENCES

1. Chen ZF, Qin QP, Qin JL, Zhou J, Li YL, Li N, Liu YC, Liang H. Water-soluble ruthenium(II) complexes with chiral 4-(2,3-dihydroxypropyl)-formamide oxoaporphine (FOA): *in vitro* and *in vivo* anticancer activity by stabilization of G-quadruplex DNA, inhibition of telomerase activity and induction of tumor cell apoptosis. J Med Chem. 2015; 58:4771-4789.
2. Cao R, Jia JL, Ma XC, Zhou M, Fei H. Membrane localized iridium(III) complex induces endoplasmic reticulum stress and mitochondria-mediated apoptosis in human cancer cells. J Med Chem. 2013; 56:3636-3644.
3. Schreiber E, Matthias P, Mueller MM, Schaffner W. Rapid detection of octamer binding proteins with 'mini-extracts' prepared from a small number of cells. Nucleic Acids Res. 1989; 17:6419.
4. Chong L, van Steensel B, Broccoli D, Erdjument-Bromage H, Hanish J, Tempst P, de Lange T. A human telomeric protein. Science. 1995; 270:1663-1667.
5. Broccoli D, Smogorzewska A, Chong L, de Lange T. Human telomeres contain two distinct Myb-related proteins, TRF1 and TRF2. Nat Genet. 1997; 17:231-235.
6. Chen ZF, Qin QP, Qin JL, Liu YC, Huang KB, Li YL, Meng T, Zhang GH, Peng Y, Luo XJ, Liang

H. Stabilization of G-quadruplex DNA, inhibition of telomerase activity and tumor cell apoptosis of organoplatinum(II) complexes with oxoisoaporphine. J Med Chem. 2015; 58:2159-2179.

7. Moore MJ, Schultes CM, Cuesta J, Cuenca F, Gunaratnam M, Tanious FA, Wilson D, Neidle S. Trisubstituted acridines as G-quadruplex telomere targeting agents. Effects of extensions of the 3, 6-and 9-side chains on quadruplex binding, telomerase activity, and cell proliferation. J Med Chem. 2006; 49:582-599.

8. Reed JE, Arnal AA, Neidle S, Vilar R. Stabilization of G-quadruplex DNA and inhibition of telomerase activity by square-planar nickel(II) complexes. J Am Chem Soc. 2006; 128:5992-5993.

9. Zou HH, Wang L, Long ZX, Qin QP, Song ZK, Xie T, Zhang SH, Liu YC, Lin B, Chen ZF. Preparation of 4-([2,2′:6′,2″-terpyridin]-4′-yl)-N, N-diethylaniline Ni^{II} and Pt^{II} complexes and exploration of their *in vitro* cytotoxic activities. Eur J Med Chem. 2016; 108:1-12.

10. Xu L, Chen X, Wu J, Wang J, Ji LN, Chao H. Dinuclear ruthenium(II) complexes that induce and stabilise G-quadruplex DNA. Chem Eur J. 2015; 21:4008-4020.

11. Mikami-Terao Y, Akiyama M, Yuza Y, Yanagisawa T, Yamada O, Yamada H. Antitumor activity of G-quadruplex-interactive agent TMPyP4 in K562 leukemic cells. Cancer Lett. 2008; 261:226-234.

12. Chalfie M, Tu Y, Euskirchen G, Ward WW, Prasher DC. Green fluorescent protein as a marker for gene expression. Science. 1994; 263:802-805.

13. He TC, Sparks AB, Rago C, Hermeking H, Zawel L, Da Costa LT, Morin PJ, Vogelstein B, Kinzler KW. Identification of c-MYC as a target of the APC pathway. Science. 1998; 281:1509-1512.

14. Sun J, Blaskovich M, Knowles D, Qian Y, Ohkanda J, Bailey R, Hamilton A, Sebti S. Antitumor efficacy of a novel class of non-thiol-containing peptidomimetic inhibitors of farnesyltransferase and geranylgeranyltransferase I. Cancer Res. 1999; 59:4919-4926.

15. Sampson PB, Liu Y, Forrest B, Cumming G, Li SW, Patel NK, Edwards L, Laufer R, Feher M, Ban F, Awrey DE, Mao G, Plotnikova O, et al. The discovery of Polo-Like Kinase 4 Inhibitors: identification of (1R,2S)-2-(3-((E)-4-(((cis)-2,6-Dimethylmorpholino)methyl)styryl)-1H-indazol-6-yl)-5′-methoxyspiro[cyclopropane-1,3′-indolin]-2′-one (CFI-400945) as a potent, orally active antitumor agent. J Med Chem. 2015; 58:147-169.

16. Palanimuthu D, Shinde S V, Somasundaram K, Samuelso AG. *In vitro* and *in vivo* anticancer activity of copper bis (thiosemicarbazone) complexes. J Med Chem. 2013; 56:722-734.

17. Zhou HY, Dong FQ, Du XL, Zhou ZK, Huo HR, Wang WH, Zhan HD, Dai YF, Meng J, Sui YP, Li J, Sui F, Zhai YH. Antitumor activities of biscoumarin and dihydropyran derivatives. Bioorg Med Chem Lett. 2016; 26:3876-3880.

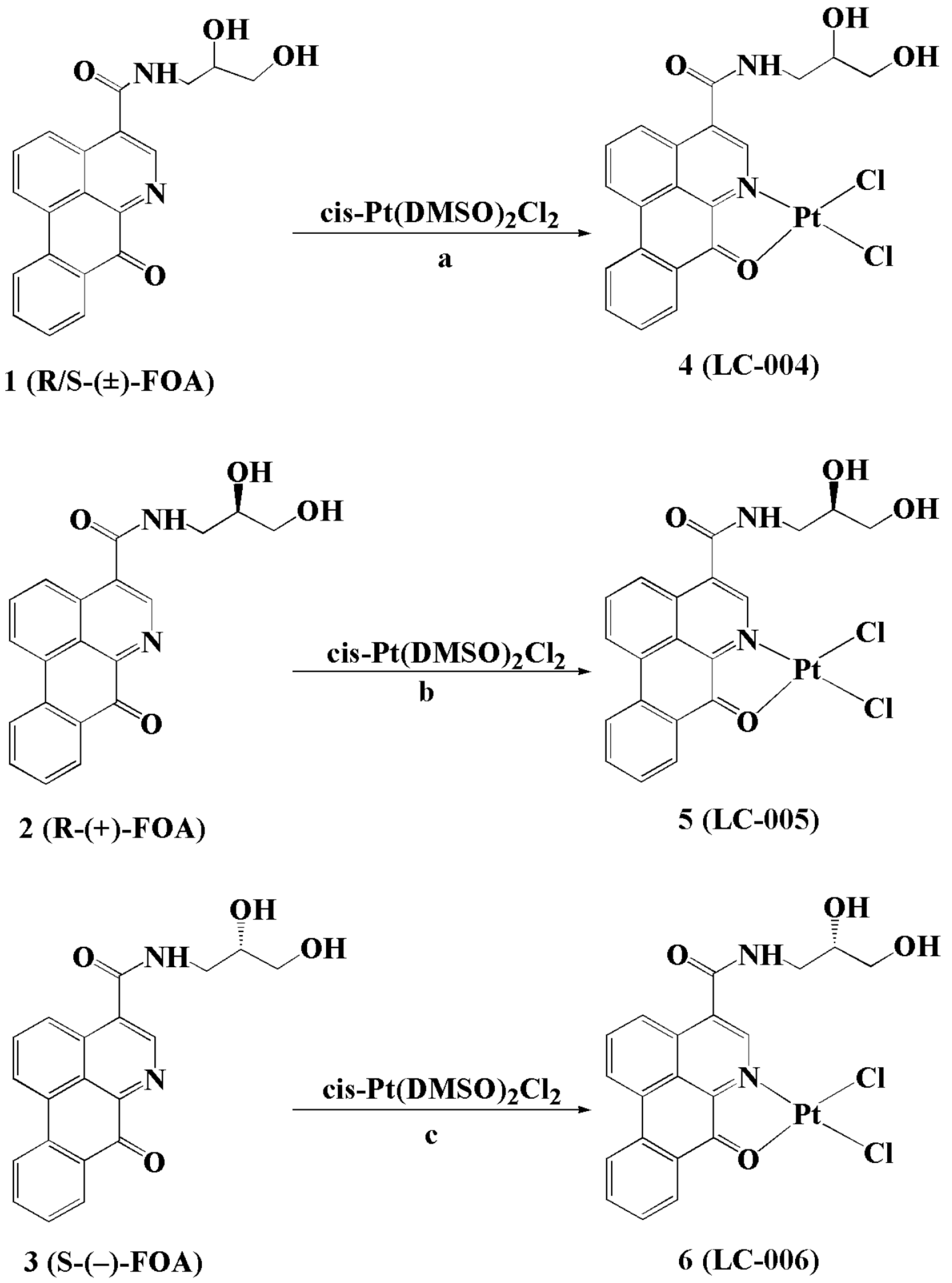

Supplementary Figure 1: Synthetic routes of the platinum(II) complexes 4–6. Reagents and solvents are the following: (a–c) methanol/water (3:1) (80 °C).

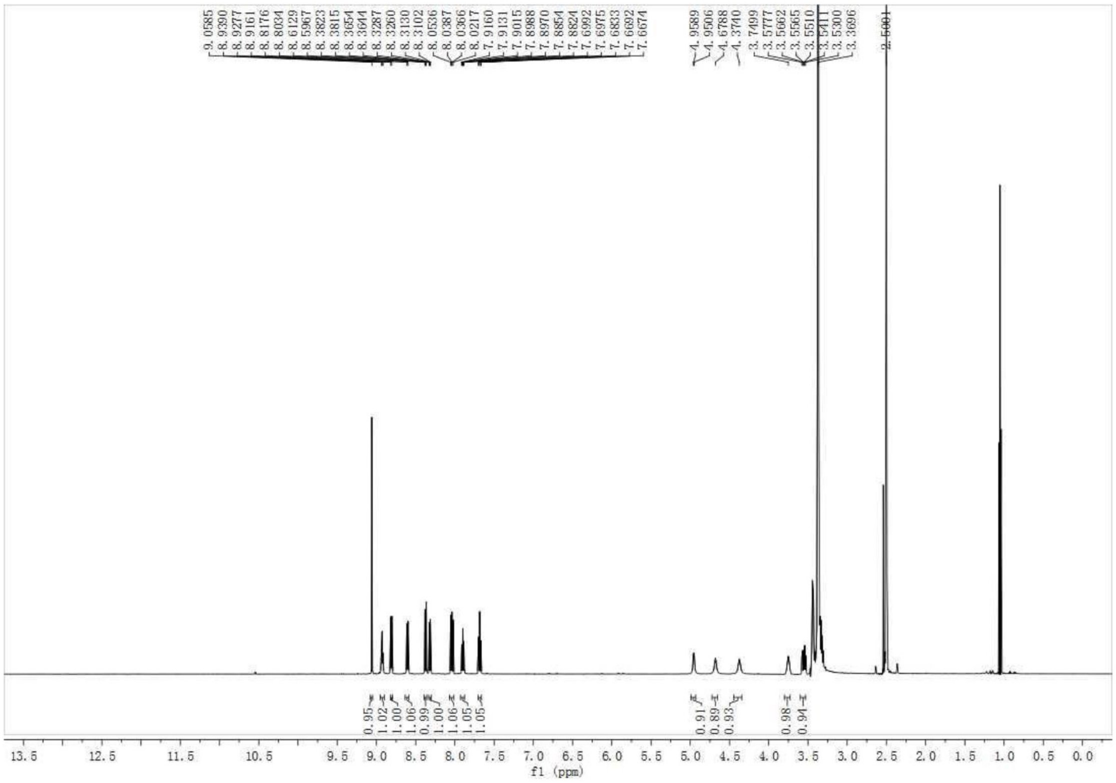

Supplementary Figure 2:^{1}**H NMR (500 MHz, DMSO-d**$_{6}$**) for 5.**

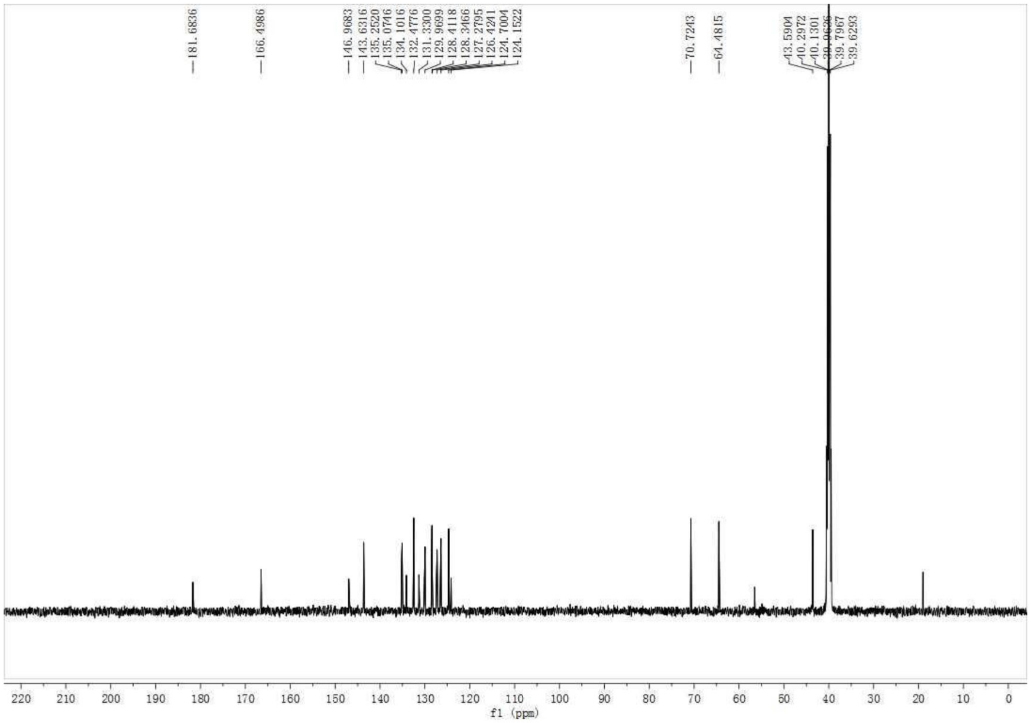

Supplementary Figure 3:^{13}**C NMR (125 MHz, DMSO-d**$_{6}$**) for 4.**

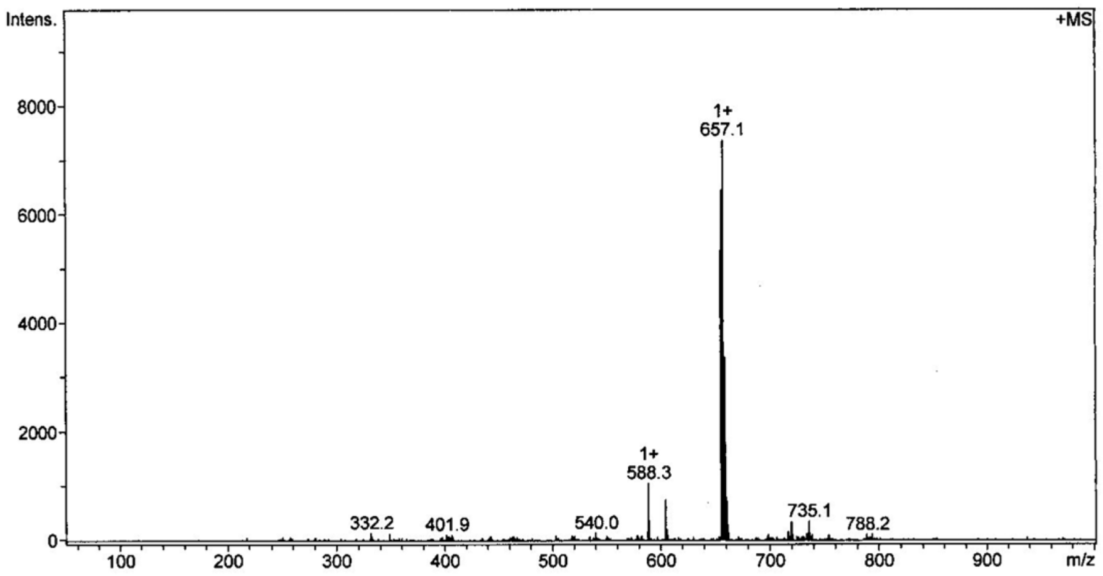

Supplementary Figure 4: MS-EI spectra of 4.

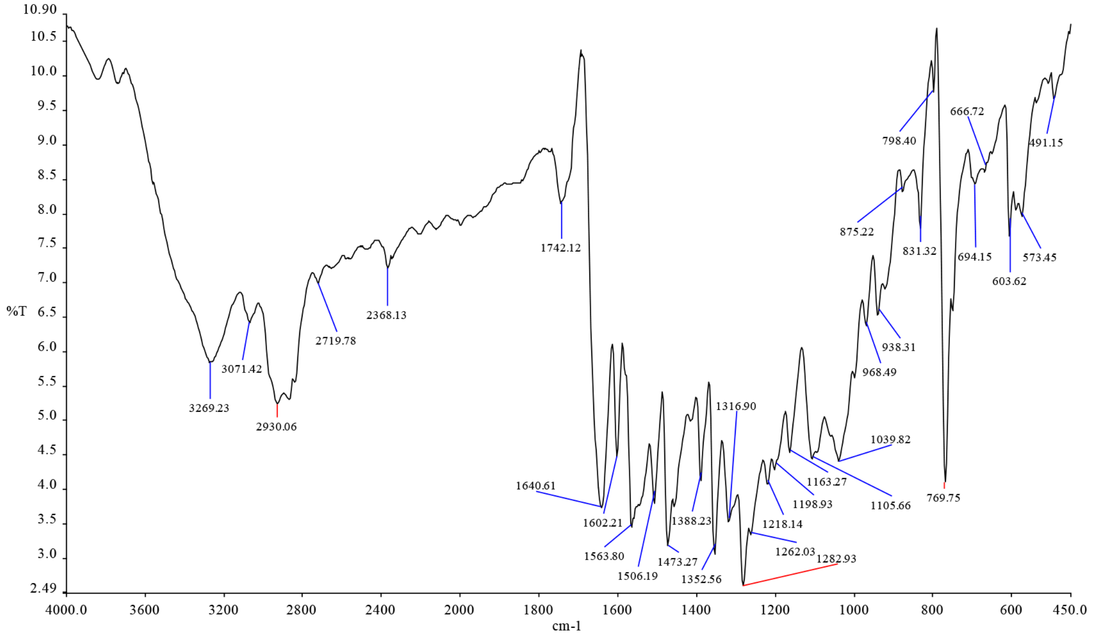

Supplementary Figure 5: IR (KBr) spectra of 4.

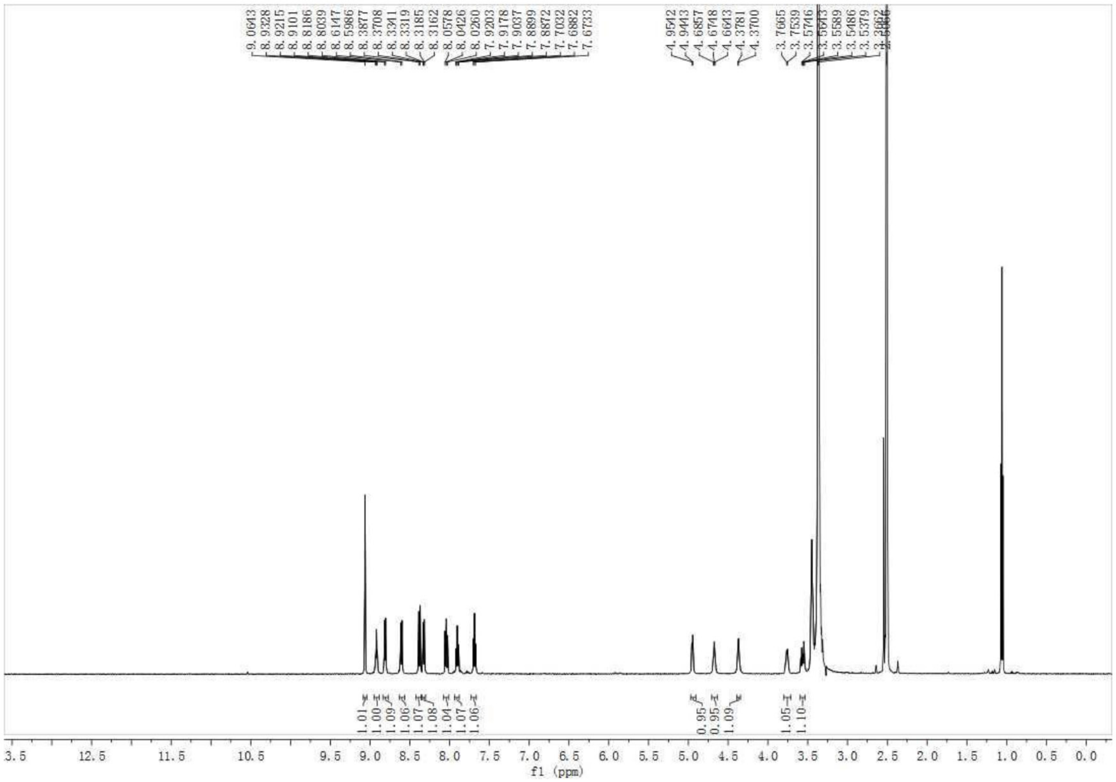

Supplementary Figure 6:^{1}H NMR (500 MHz, DMSO-d_6) for 5.

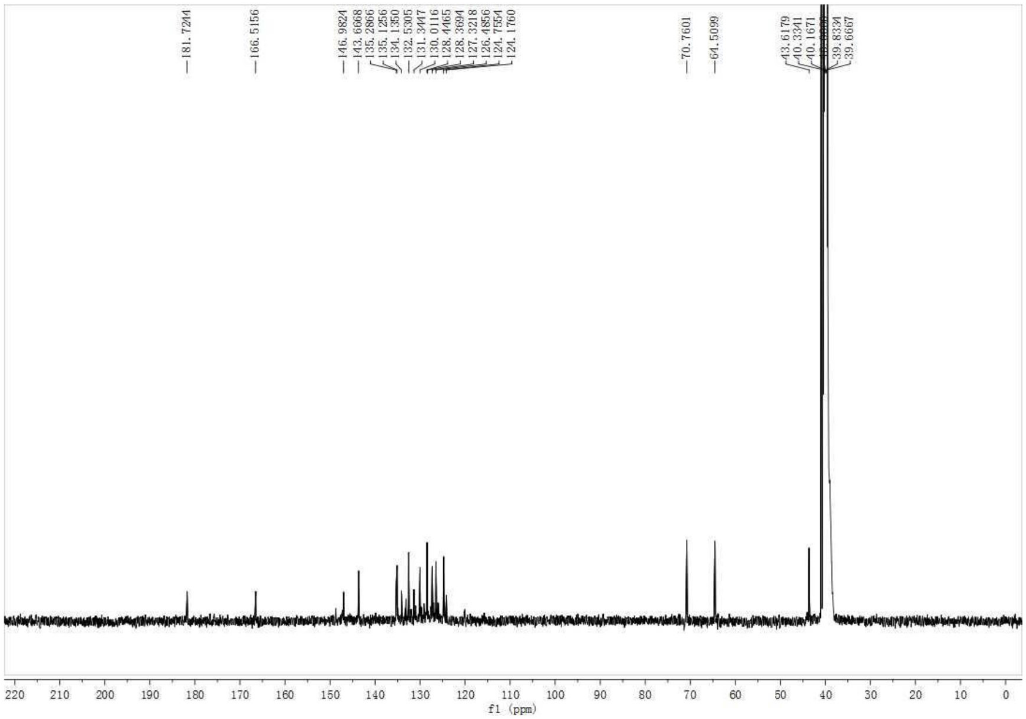

Supplementary Figure 7:^{13}C NMR (125 MHz, DMSO-d_6) for 5.

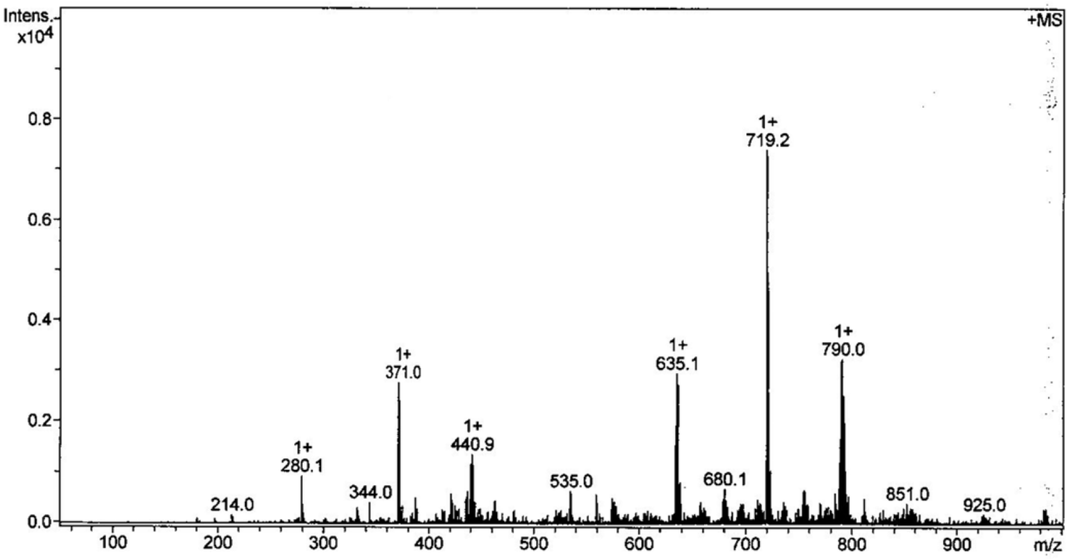

Supplementary Figure 8: MS-EI spectra of 5.

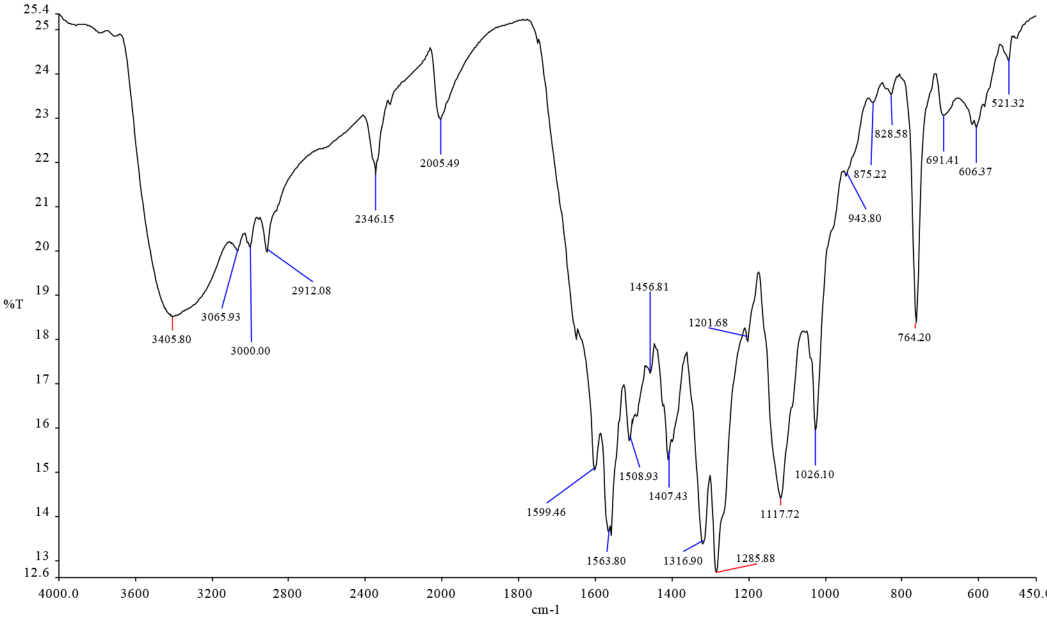

Supplementary Figure 9: IR (KBr) spectra of 5.

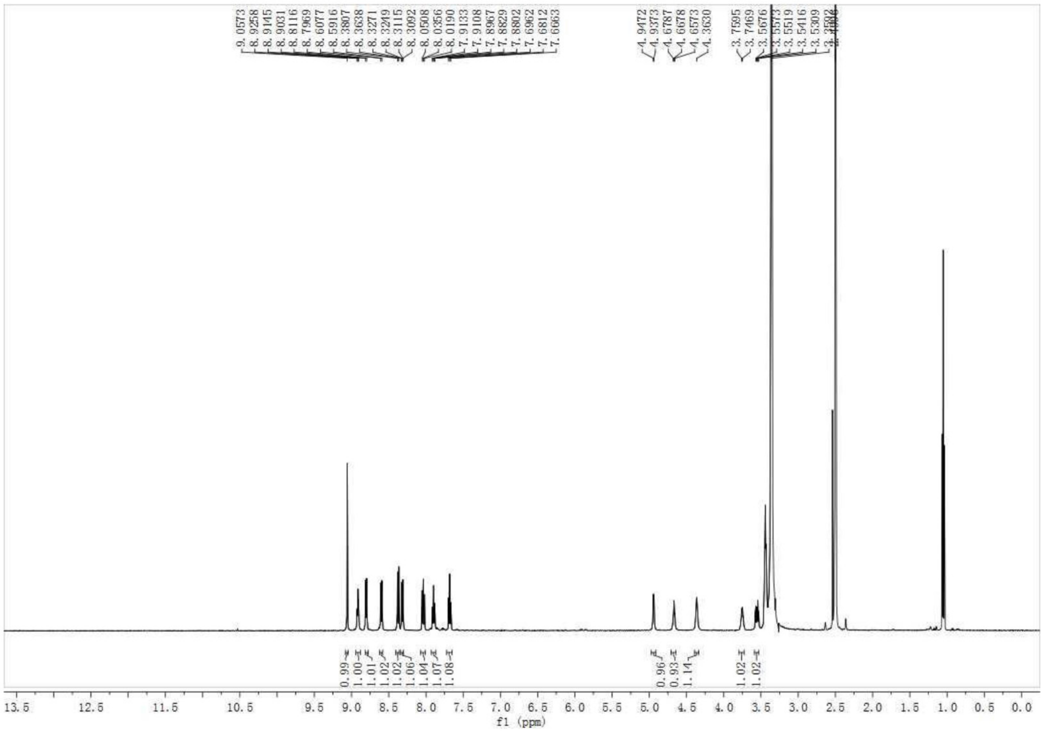

Supplementary Figure 10:^{1}H NMR (500 MHz, DMSO-d$_6$) for 6.

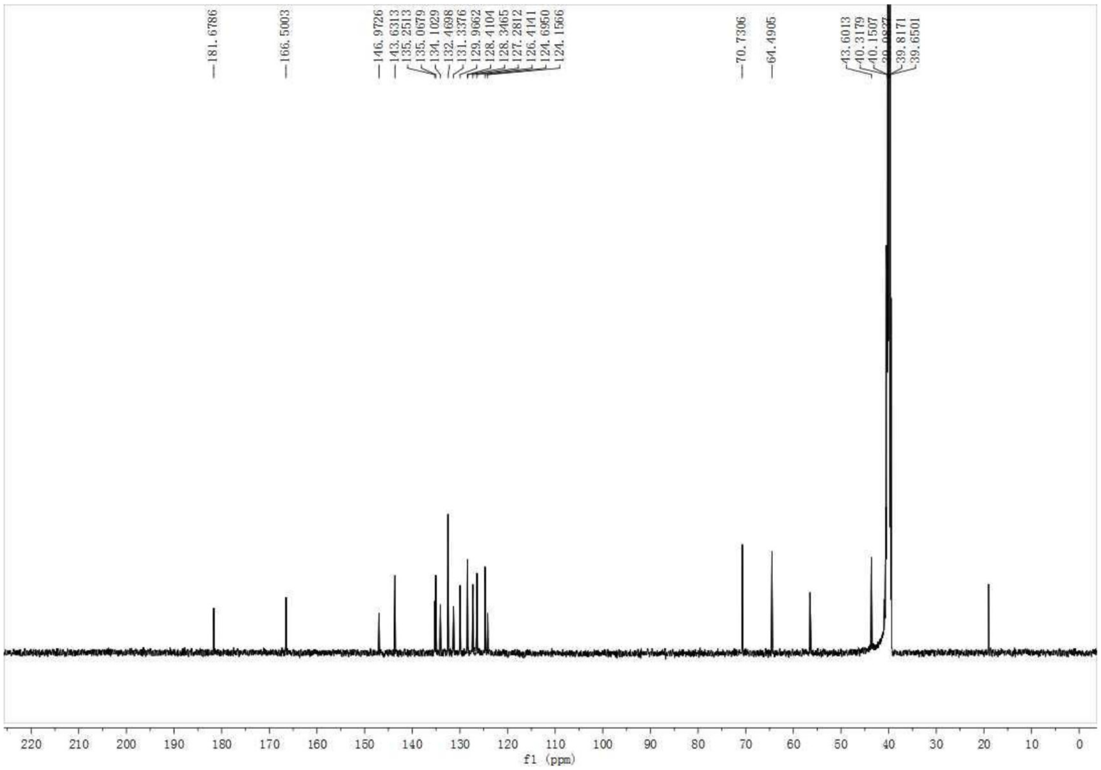

Supplementary Figure 11:^{13}C NMR (125 MHz, DMSO-d$_6$) for 6.

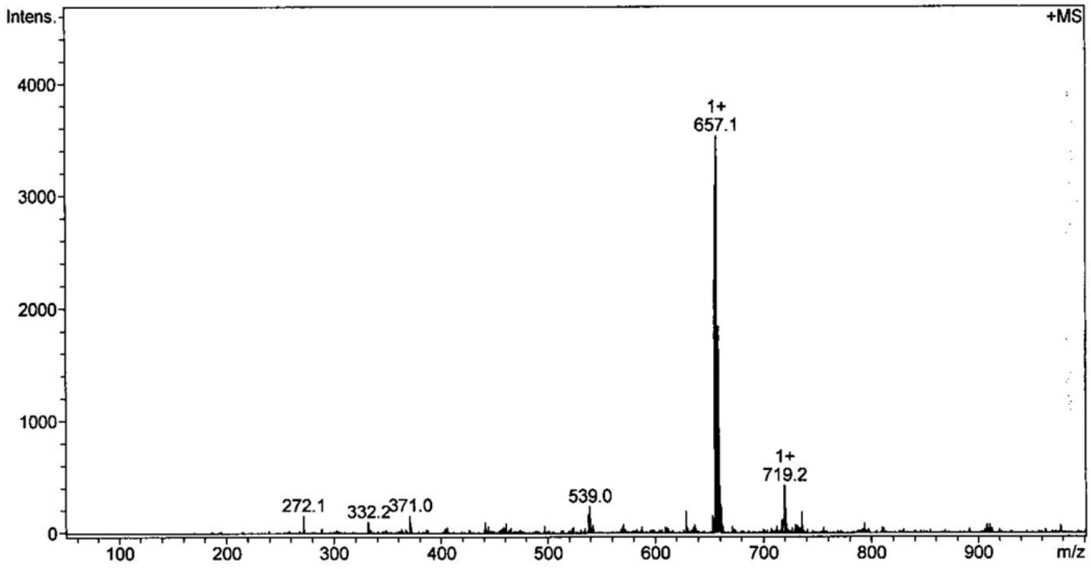

Supplementary Figure 12: MS-EI spectra of 6.

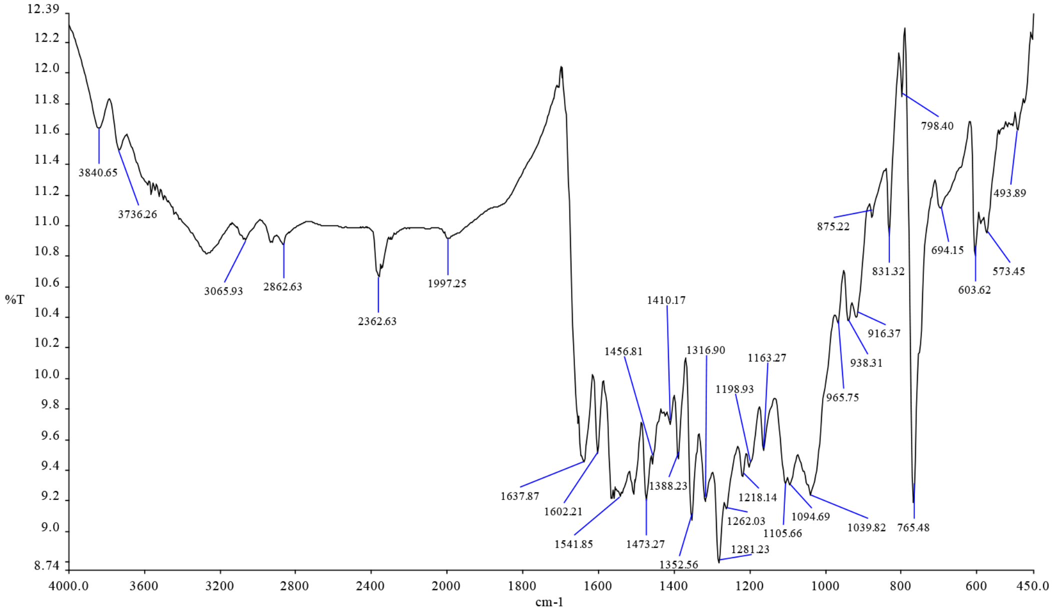

Supplementary Figure 13: IR (KBr) spectra of 6.

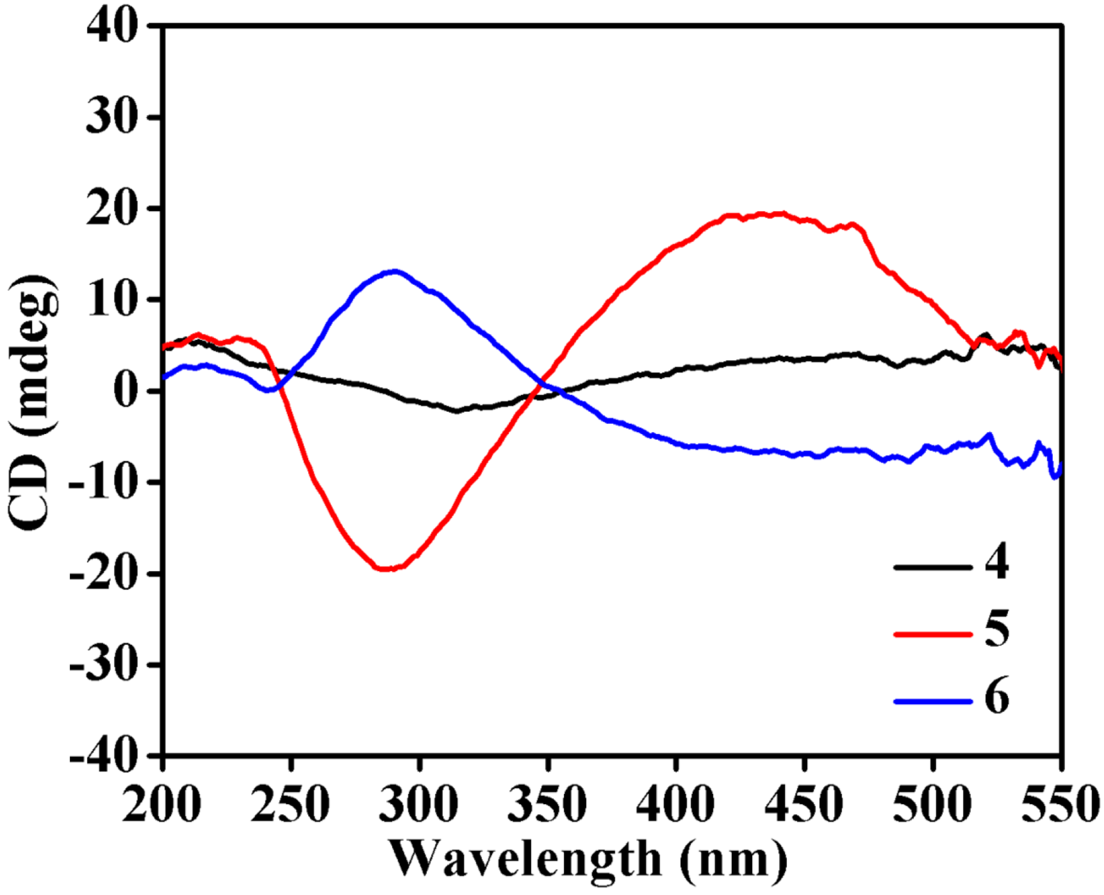

Supplementary Figure 14: CD spectra of 4-6 in DMSO/Tris (v/v = 2:1) solution (1.0×10^{-3} M), respectively.

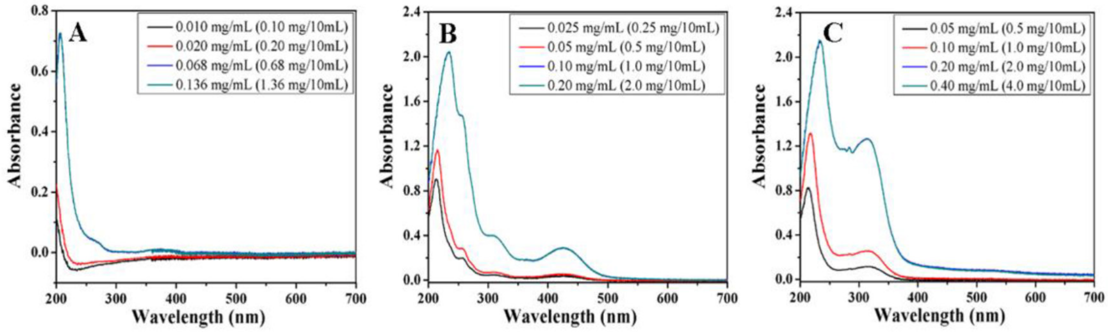

Supplementary Figure 15: UV-Vis absorption spectra of 4 **(A)**, 5 **(B)**, and 6 **(C)** in distilled water at room temperature, respectively. (Solution by UV-Vis measurement after diluted 10 times)

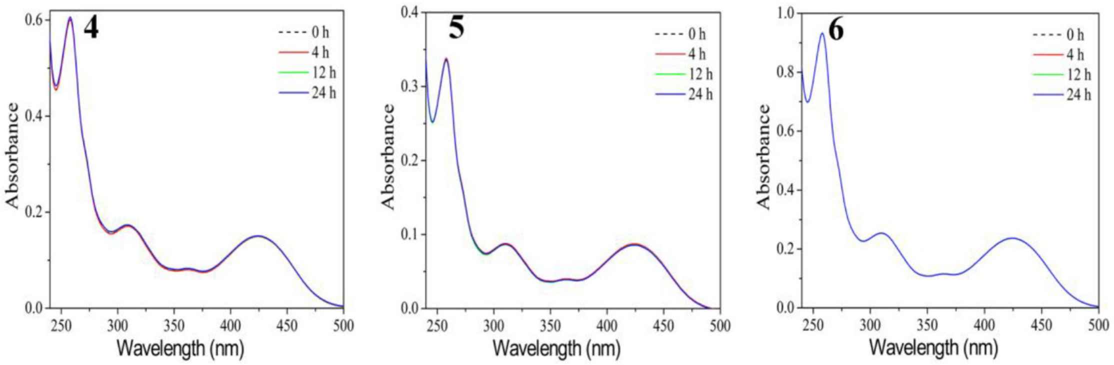

Supplementary Figure 16: UV-Vis absorption spectra of 4-6 in Tris-KCl-HCl solution (3.0×10^{-5} M) in the time course 0 h, 4 h, 12 h and 24 h, respectively.

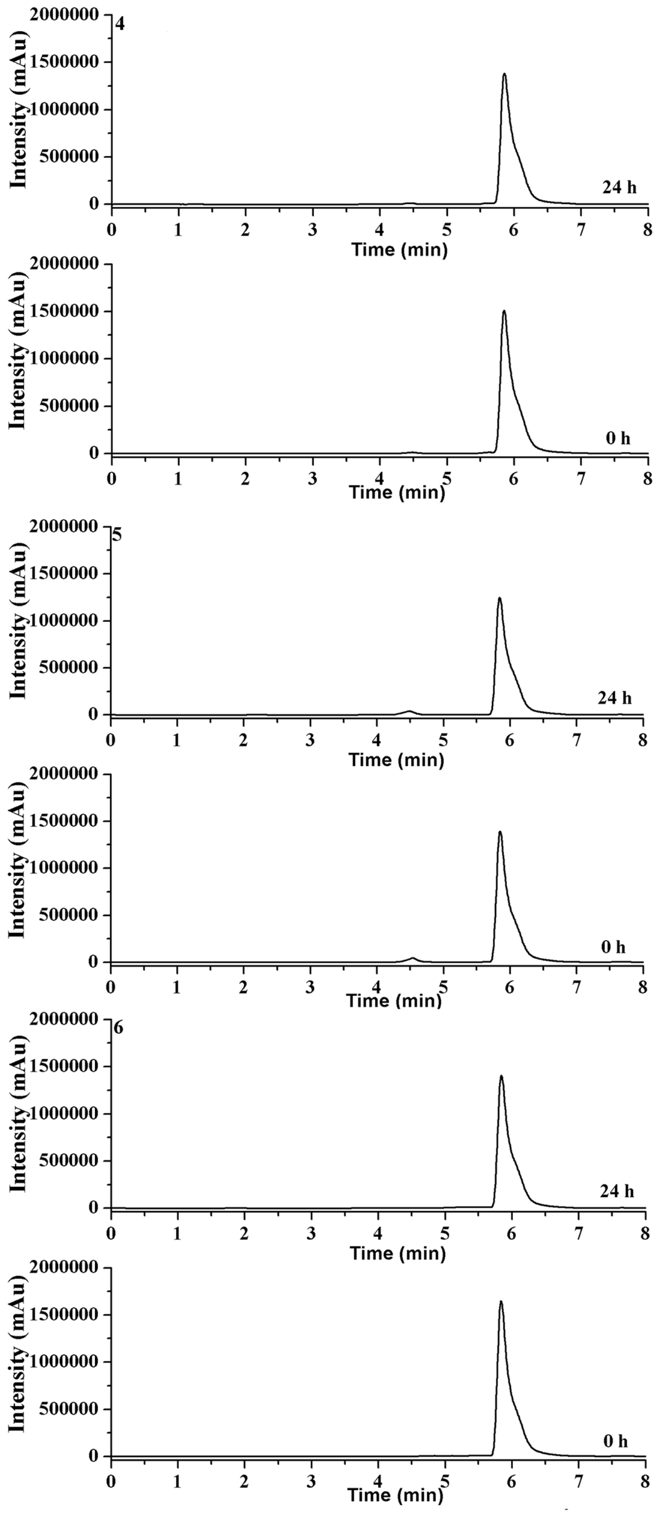

Supplementary Figure 17: HPLC spectra for 4-6 in DMSO (2.0×10^{-3} M) with 0 h and 24 h. Column: Inertsustain C18 column (LC-20AT, SPD-20A HPLC COLUMN, 150mm×5.0μm I.D.). Column temperature: 40ºC. Mobile phase: methanol/H_2O containing 0.01% TFA (88:12 methanol/H_2O). Flow rate: 1.0 mL/min. Injection volume: 3.0×10^{-4} M.

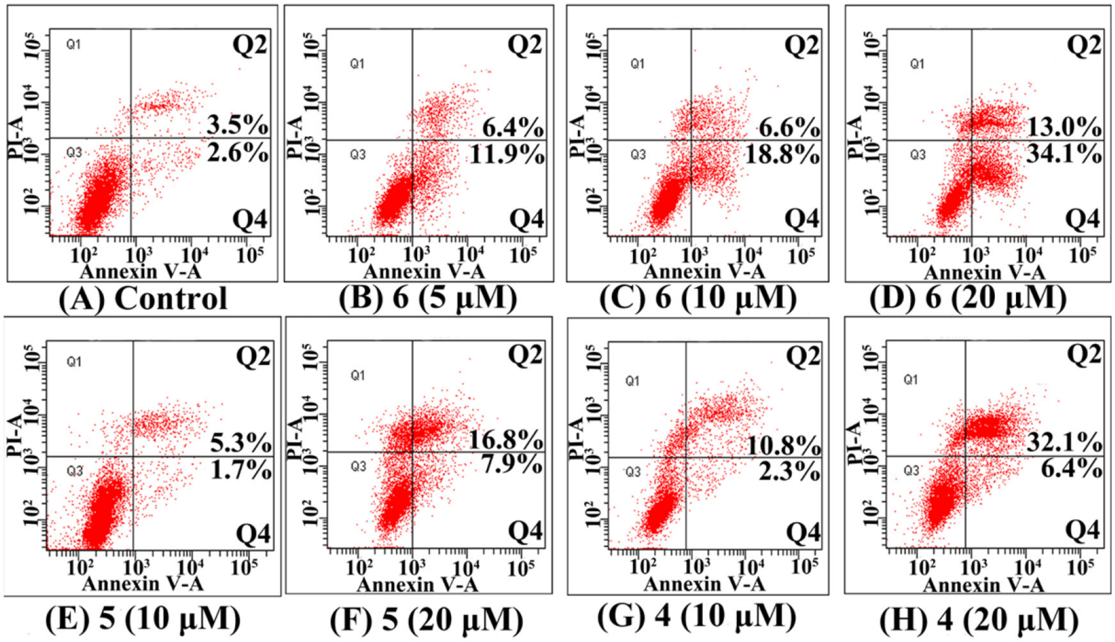

Supplementary Figure 18: Flow cytometric assays showing the effect of treatment of BEL-7404 cells with 6 **(B-D)**, 4 **(E and F)** and 5 **(G and H)** for 24 h on cell apoptosis at early- and late-stages as compared with those of the control group cells **(A)**, respectively.

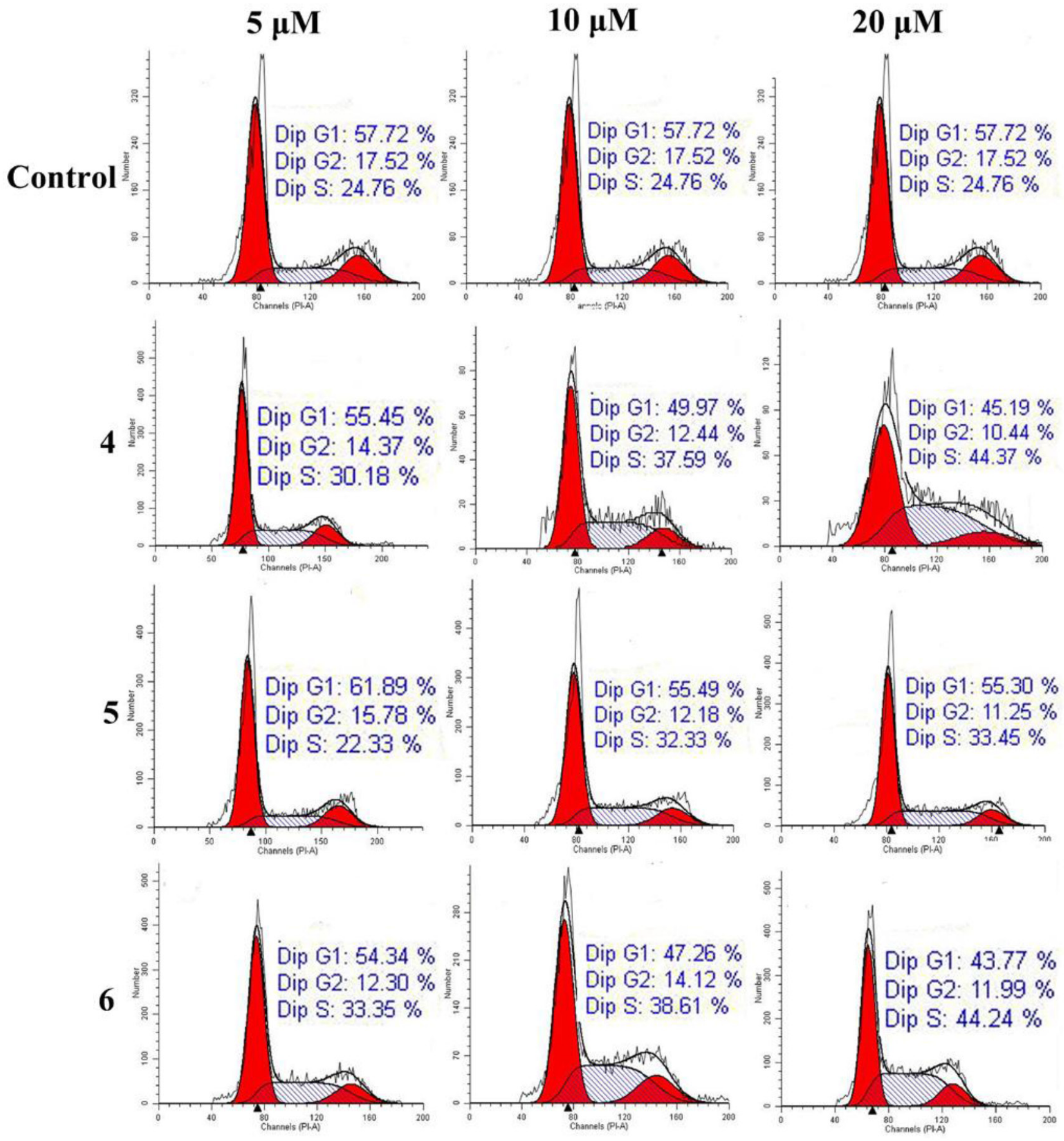

Supplementary Figure 19: Effect of cell cycle of BEL-7404 treated with 4-6 for 48 h comparing with untreated cells. The percentages of cells in the different phases of cell cycle are reported inside the relative histogram.

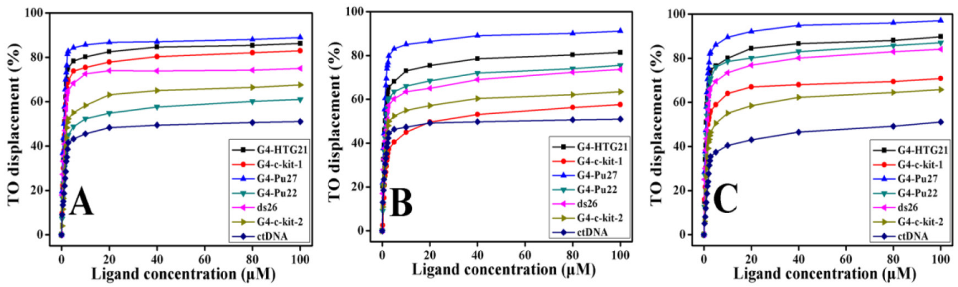

Supplementary Figure 20: FID results onto 4 **(A)**, 5 **(B)** and 6 **(C)** toward DNA in sodium cacodylate buffer solution.

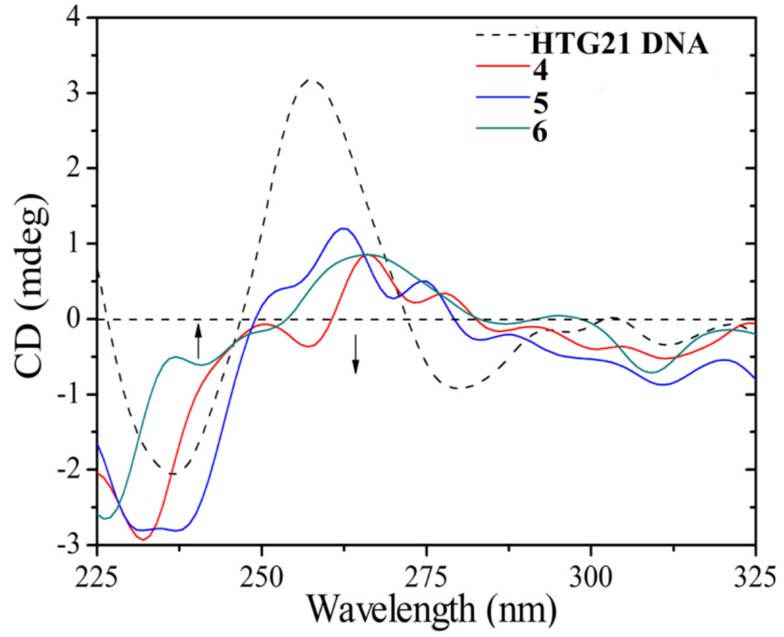

Supplementary Figure 21: CD spectra of Human Telomeric DNA (10 μM) in the absence of K^+ (10 mM pH 7.35 Tris-HCl) upon addition of compounds (20 μM).

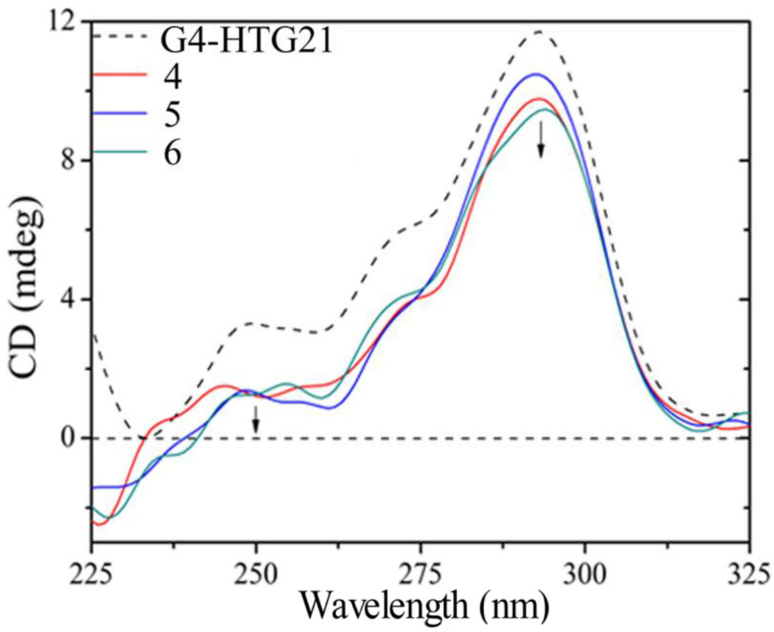

Supplementary Figure 22: CD spectra of Human Telomeric G-quadruplex DNA (10 µM) in the presence of K^+ (TBS buffer, containing 1% DMSO, 100 mM KCl, and 10 mM pH 7.35 Tris-HCl) upon addition of compounds (20 µM).

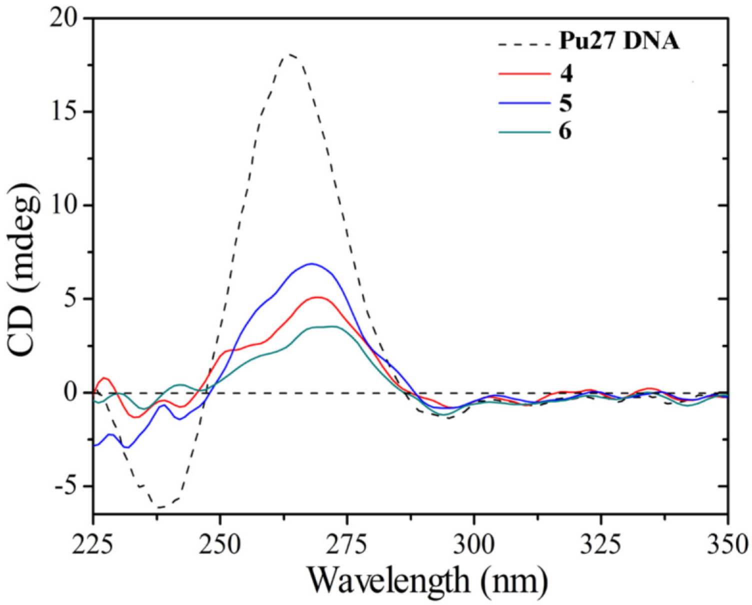

Supplementary Figure 23: CD spectra of Pu27 DNA (10 µM) in the absence of K^+ upon addition of compounds (20 µM).

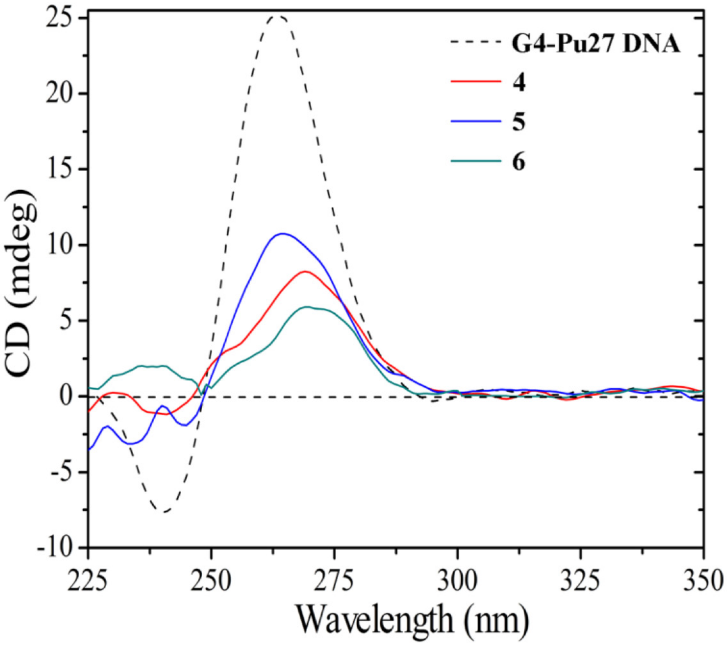

Supplementary Figure 24: CD spectra of G-quadruplex Pu27 DNA (10 μM) in the presence of K^+ upon addition of compounds (20 μM).

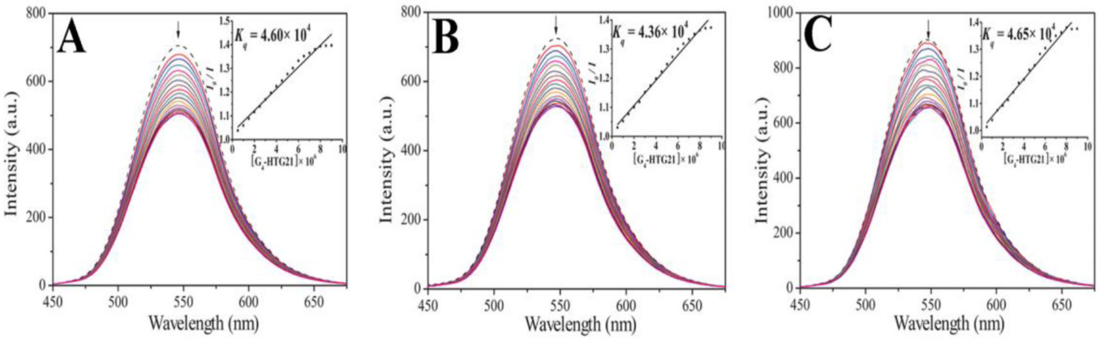

Supplementary Figure 25: Fluorescence emission spectra of compounds (20 μM) in 100 mM K^+ solution in the absence and presence of human telomeric G-quadruplex DNA with 4 **(A)**, 5 **(B)** and 6 **(C)**, respectively.

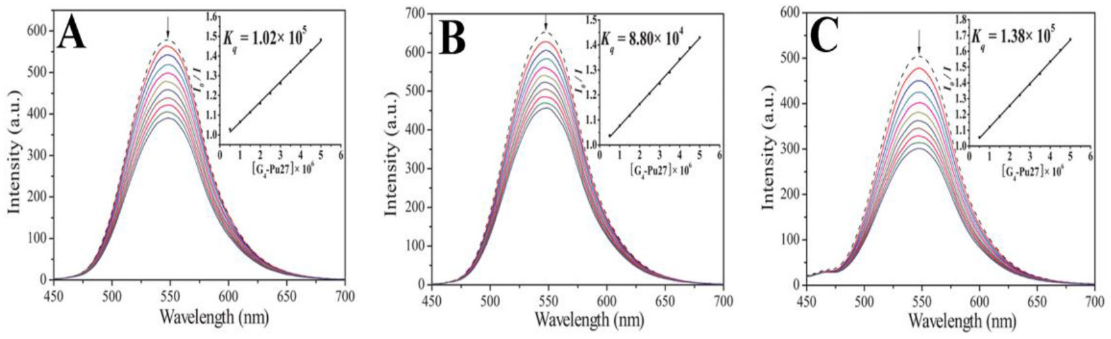

Supplementary Figure 26: Fluorescence emission spectra of compounds (20 μM) in 100 mM K^+ solution in the absence and presence of G-quadruplex Pu27 with 4 **(A)**, 5 **(B)** and 6 **(C)**, respectively.

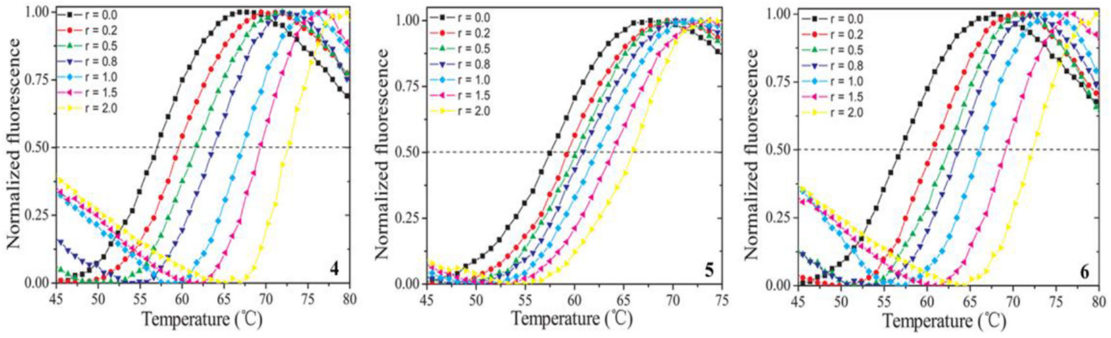

Supplementary Figure 27: FRET melting curves for experiments carried out with FPu18T (1.0 μM in 10 mM Tris-HCl, 100 mM KCl, pH 7.35) with: **(A)** 4; **(B)** 5; **(C)** 6. r = [each compound]/[F21T].

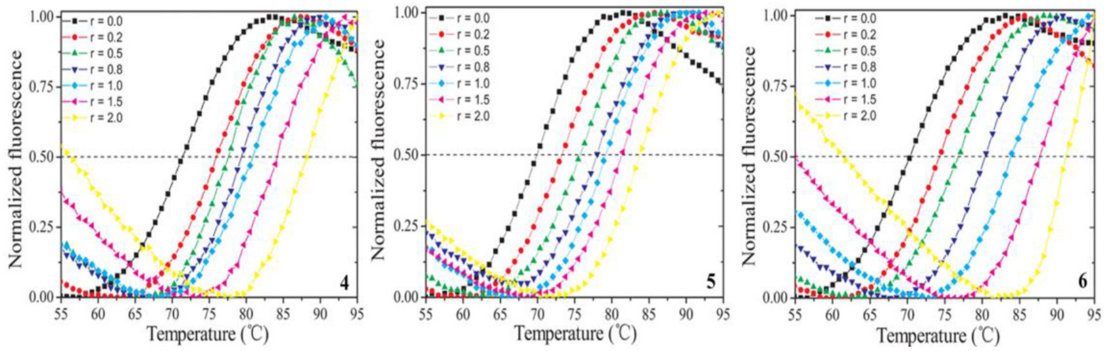

Supplementary Figure 28: FRET melting curves for experiments carried out with FPu18T (1.0 μM in 10 mM Tris-HCl, 100 mM KCl, pH 7.35) with: **(A)** 4; **(B)** 5 and **(C)** 6 binding to FPu18T. r = [each compound]/[FPu18T].

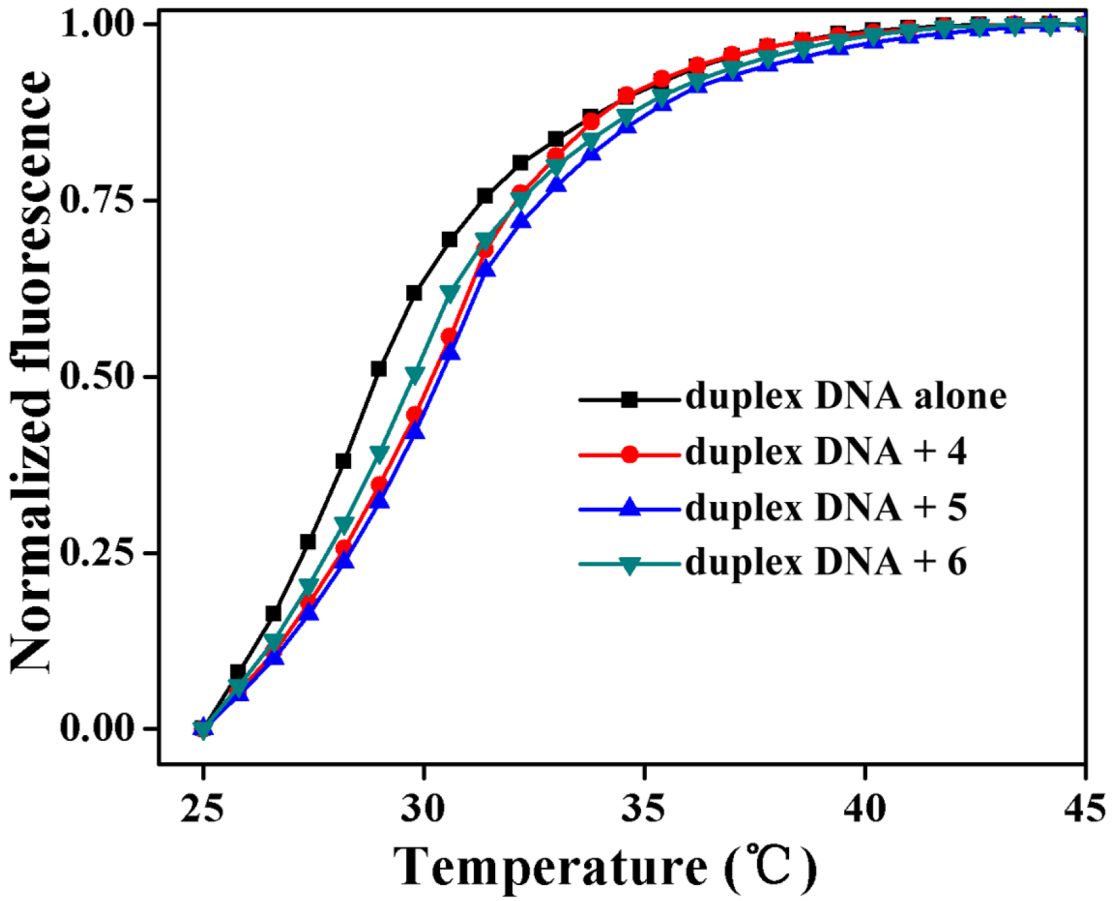

Supplementary Figure 29: FRET melting curves for experiments carried out with duplex DNA (F32T+H20M, 1.0 μM in 10 mM Tris-HCl, 100 mM KCl, pH 7.35) with 4-6 binding to duplex DNA, respectively.

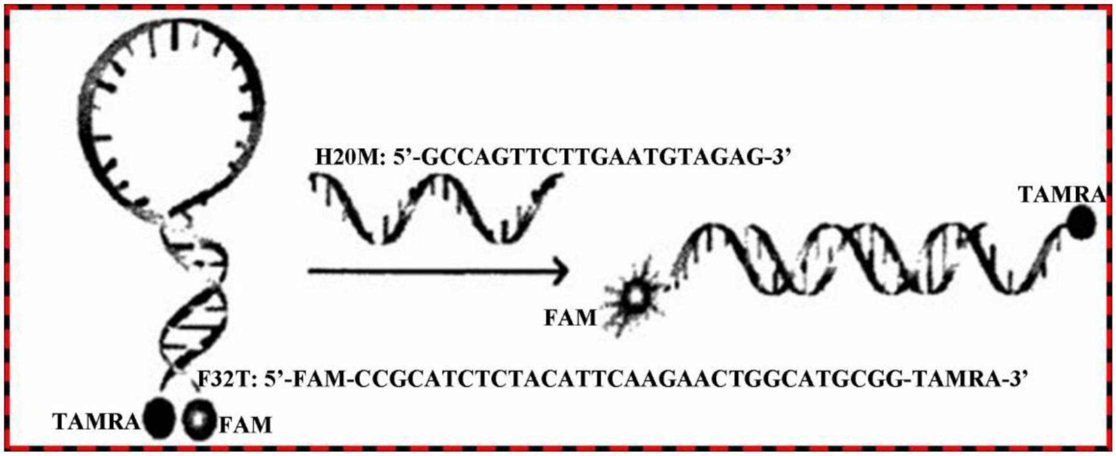

Supplementary Figure 30: Duplex DNA (F32T: 5'-FAM-CCGCATCTCTACATTCAAGAACTGGCATGCGG-TAMRA-3'; H20M: 5'-GCCAGTTCTTGAATGTAGAG-3').

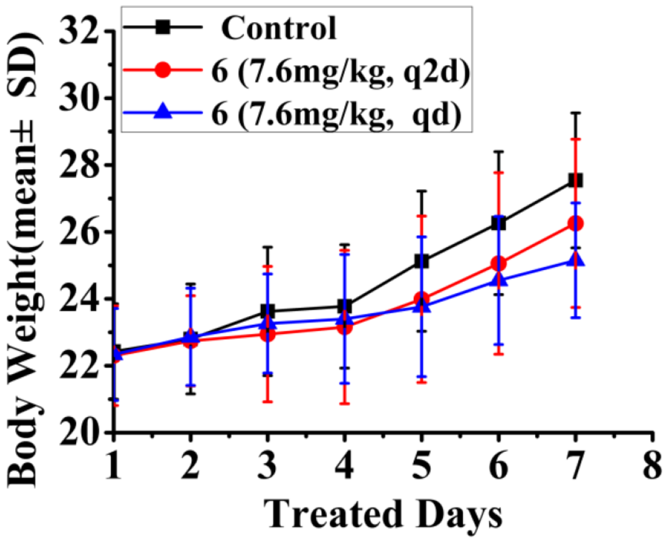

Supplementary Figure 31: The average body weight of KM mice in treated with complex 6.

Supplementary Table 1: Abbreviations, cell lines and DNA oligomers of used in this work.

See Supplementary File 1

Supplementary Table 2: Inhibitory rates (%) of 1-6, *cis*-Pt(DMSO)$_2$Cl$_2$ and cisplatin toward six tumor cell lines and normal liver HL-7702 for 48 h

Compounds	BEL-7404	A549	MGC80-3	HeLa	Hep-G2	BEL-7402	HL-7702
**1**[a]	51.9 ± 2.7	48.4 ± 1.6	24.9 ± 2.2	33.6 ± 1.3	27.8 ± 0.8	38.9 ± 1.6	15.2 ± 0.6
**2**[a]	50.0 ± 0.6	47.9 ± 0.9	19.6 ± 0.8	26.8 ± 1.2	23.6 ± 2.3	22.3 ± 1.1	30.6 ± 2.1
**3**[a]	64.8 ± 1.3	51.2 ± 3.4	28.2 ± 1.4	36.6 ± 1.5	29.3 ± 0.9	41.8 ± 2.5	27.5 ± 1.2
**4**[a]	80.0 ± 4.3	77.8 ± 0.1	24.9 ± 1.4	24.1 ± 2.1	27.6 ± 1.1	48.6 ± 1.3	31.6 ± 2.4
**5**[a]	55.9 ± 2.7	35.9 ± 2.6	19.8 ± 1.8	9.9 ± 0.7	23.3 ± 0.5	42.1 ± 0.7	37.5 ± 5.3
**6**[a]	81.3 ± 0.4	84.7 ± 2.2	26.5 ± 1.1	25.1 ± 1.4	39.3 ± 2.2	58.4 ± 2.1	33.9 ± 2.2
cis-Pt(DMSO)$_2$Cl$_2$[b]	8.0 ± 0.7	22.3 ± 0.5	30.4 ± 0.8	25.4 ± 1.2	13.8 ± 0.7	12.3 ± 0.4	No data
cisplatin[a]	54.2 ± 1.4	51.1 ± 3.4	71.4 ± 1.5	80.6 ± 0.9	60.6 ± 1.0	65.7 ± 1.2	73.6 ± 2.3

Results represent mean ± SD of at least five independent experiments. SD represents the standard deviation. [a]The concentration is 20 μM. [b]The concentration is 100 μM.

Supplementary Table 3: Inhibitory rates (%) of 1-6, *cis*-Pt(DMSO)$_2$Cl$_2$ and cisplatin toward six tumor cell lines and normal liver HL-7702 for 24 h

Compounds	BEL-7404	A549	MGC80-3	HeLa	Hep-G2	BEL-7402	HL-7702
**1**[a]	35.9 ± 0.7	32.6 ± 0.4	18.6 ± 0.3	25.6± 1.2	21.3 ± 1.9	30.5 ± 1.4	11.0 ± 1.6
**2**[a]	25.1 ± 1.2	28.9 ± 1.4	9.6 ± 0.6	18.9 ± 0.6	17.5 ± 0.4	17.0 ± 0.6	25.2 ± 0.3
**3**[a]	42.8 ± 0.4	37.2 ± 0.8	24.2 ± 0.5	31.2 ± 0.4	24.9 ± 0.8	35.1 ± 1.3	23.4 ± 0.9
**4**[a]	67.3 ± 1.3	57.6 ± 0.6	22.0 ± 1.9	18.5 ± 0.7	20.8 ± 0.3	41.2 ± 0.6	28.3 ± 1.7
**5**[a]	46.1 ± 1.1	28.9 ± 0.5	11.3 ± 0.4	8.6 ± 1.5	16.5 ± 1.4	35.6 ± 2.1	30.6 ± 2.1
**6**[a]	70.9 ± 0.7	65.3 ± 1.3	25.1 ± 0.5	21.1 ± 0.9	30.2 ± 1.6	50.5 ± 1.6	20.7 ± 1.6
cis-Pt(DMSO)$_2$Cl$_2$[b]	5.3 ± 1.5	15.1 ± 1.7	10.2 ± 1.3	19.8 ± 0.7	10.1 ± 0.4	8.6 ± 2.4	No data
cisplatin[a]	45.5 ± 2.1	48.6 ± 0.4	61.5 ± 1.2	64.3 ± 1.4	51.6 ± 0.6	55.4 ± 0.9	59.6 ± 1.2

Results represent mean ± SD of at least five independent experiments. SD represents the standard deviation. [a]The concentration is 20 µM. [b]The concentration is 100 µM.

Supplementary Table 4: Complexes 1-6 and cisplatin towards six cancer-cell lines and one HL-7702 cell line for 48 h

compds.	MGC80-3	A549	BEL-7404	Hep-G2	BEL-7402	HeLa	HL-7702[c]
**1**[a]	32.4±0.8	20.2±0.5	17.2±0.1	32.9±0.5	30.4±1.5	25.7±2.2	>100 (>5.8)
**2**[a]	45.8±2.8	24.1±0.4	20.5±0.1	38.9±1.3	68.6±0.8	28.5±0.3	>100 (>4.9)
**3**[a]	35.7±2.9	20.4±0.3	15.4±0.2	24.9±0.4	28.5±2.4	22.9±0.7	81.4±2.7 (5.3)
**4**	32.1 ± 1.8	9.1 ± 0.6	8.7 ± 0.1	29.1 ± 0.8	20.7 ± 0.9	38.1 ± 1.5	58.5 ± 5.5 (6.7)
**5**	37.4 ± 1.2	17.2 ± 0.1	14.5 ± 0.5	35.7 ± 1.5	38.4 ± 1.4	64.2 ± 0.2	55.8 ± 3.1 (3.9)
**6**	31.5 ± 1.7	8.2 ± 0.5	7.9 ± 0.3	17.7 ± 1.2	15.3 ± 2.2	31.8 ± 0.7	84.2 ± 1.2 (10.7)
Cisplatin[b]	5.4 ± 0.5	17.0 ± 2.3	15.8 ± 0.7	9.5 ± 0.4	13.5 ± 0.9	5.8 ± 0.9	15.6 ± 0.3 (1.0)

[a] The data cited from reference [1]. [b] Cisplatin was dissolved by Cao and Fei method [2]. In brief, cisplatin was dissolved at a concentration of 1.0 mM in 0.154 M NaCl. [c] The selectivity index factor, defined as IC_{50} (normal HL-7702 cells)/IC_{50} (BEL-7404 tumor cells), was given in parentheses [17].

Supplementary Table 5: Complexes 1-6 and cisplatin towards six cancer-cell lines and one HL-7702 cell line for 24 h

Compds.	MGC80-3	A549	BEL-7404	Hep-G2	BEL-7402	HeLa	HL-7702[b]
**1**	87.2 ± 0.5	45.8 ± 0.3	41.2 ± 0.6	73.5±2.0	54.9 ± 1.2	69.3±1.3	141.3 ± 1.7 (3.4)
**2**	158.1 ± 0.6	67.2 ± 1.5	72.3 ± 1.0	90.6±0.3	94.1±0.5	87.5±0.8	101.8 ± 0.4 (1.4)
**3**	78.2 ± 0.7	36.4 ± 1.0	30.1 ± 0.7	70.1 ± 0.8	45.8 ±1.4	43.2±0.4	105.3 ± 1.0 (3.5)
**4**	80.6 ± 2.0	18.6 ± 0.5	12.5 ± 1.1	84.6 ± 0.4	32.5 ± 0.5	90.5 ± 0.7	85.4 ± 1.5 (6.8)
**5**	135.1 ± 0.5	56.3 ± 0.7	22.5 ± 1.3	93.4 ± 1.2	42.3 ± 1.9	168.6 ± 1.4	80.1 ± 1.8 (3.6)
**6**	72.8 ± 0.4	15.3 ± 1.2	10.1 ± 0.6	50.1 ± 1.8	20.6 ± 1.5	78.2 ± 0.9	118.9 ± 1.5 (11.8)
Cisplatin[a]	16.3 ± 1.3	21.3 ± 0.7	24.8 ± 1.8	19.5 ± 0.5	17.5 ± 0.6	15.8 ± 1.2	19.8 ± 1.3 (0.8)

[a] Cisplatin was dissolved by Cao and Fei method [2]. In brief, cisplatin was dissolved at a concentration of 1.0 mM in 0.154 M NaCl. [b] The selectivity index factor, defined as IC_{50} (normal HL-7702 cells)/IC_{50} (BEL-7404 tumor cells), was given in parentheses [17].

Supplementary Table 6: FID assay (DC_{50}, μM) forligands 1-3 and theirchiral platinum(II) complexes 4-6

	1[a]	2[a]	3[a]	4	5	6
$^{Pu27}DC_{50}$	1.63	1.77	1.43	1.01	0.89	0.82
($^{ctDNA}DC_{50}/^{Pu27}DC_{50}$)	(39.24)	(22.59)	(62.81)	(75.11)	(71.56)	(110.67)
$^{HTG21}DC_{50}$	2.48	2.25	1.85	1.23	1.26	0.99
($^{ctDNA}DC_{50}/^{HTG21}DC_{50}$)	(25.79)	(17.78)	(48.55)	(61.68)	(50.54)	(90.65)
$^{c\text{-}kit\text{-}2}DC_{50}$	>100	>100	22.94	2.48	3.08	4.78
($^{ctDNA}DC_{50}/^{c\text{-}kit\text{-}2}DC_{50}$)	(<0.64)	(<0.40)	(3.92)	(30.59)	(20.68)	(18.98)
$^{c\text{-}kit\text{-}1}DC_{50}$	66.35	>100	2.45	1.45	23.89	1.75
($^{ctDNA}DC_{50}/^{c\text{-}kit\text{-}1}DC_{50}$)	(0.96)	(<0.40)	(36.66)	(52.32)	(2.67)	(51.85)
$^{Pu22}DC_{50}$	33.97	57.62	25.75	7.41	1.74	1.14
($^{ctDNA}DC_{50}/^{Pu22}DC_{50}$)	(1.88)	(0.69)	(3.49)	(10.24)	(36.60)	(79.59)
$^{ds26}DC_{50}$	12.05	14.41	7.88	1.65	2.23	1.39
($^{ctDNA}DC_{50}/^{ds26}DC_{50}$)	(5.31)	(2.77)	(11.40)	(45.98)	(28.56)	(65.27)
$^{ctDNA}DC_{50}$	63.96	39.98	89.82	75.87	63.69	90.73
($^{ctDNA}DC_{50}/^{ctDNA}DC_{50}$)	(1.00)	(1.00)	(1.00)	(1.00)	(1.00)	(1.00)

[a] The data cited from reference [1].

Supplementary Table 7: The changes of CD spectra of binding to G-quadruplexes by 4-6

Compd	DNA	Extent of change at 250—265 nm (%)	Extent of change at 290—295 nm (%)
**4**	HTG21 (G_4-HTG21)	73.35↓ (50.08↓)	**— (16.44↓)**
	Pu27 (G_4-Pu27)	72.16↓ (67.47↓)	—
**5**	HTG21 (G_4-HTG21)	62.38↓ (52.31↓)	**—(9.76↓)**
	Pu27 (G_4-Pu27)	62.63↓ (57.01↓)	—
**6**	HTG21 (G_4-HTG21)	73.98↓ (56.31↓)	**— (19.43↓)**
	Pu27 (G_4-Pu27)	80.88↓ (76.23↓)	—

"—"represents no obvious changes in the peaks.

Supplementary Table 8: Binding constants of 4-6 upon addition of G-quadruplex DNA

Compds	G4-DNA	Extent of quenching (%)	K_q (L·mol^{-1})
**4**	HTG21	27.34	4.60×10^4
	Pu27	32.54	1.02×10^5
**5**	HTG21	27.21	4.36×10^4
	Pu27	30.06	8.80×10^4
**6**	HTG21	28.42	4.65×10^4
	Pu27	40.31	1.38×10^5

Supplementary Table 9: ΔT_m data (°C) of G-quadruplex DNA and duplex DNA (F32T+H20M) obtained by real-PCR

Compds	DNA (1.0 µM)	Conc. of Compd	ΔT_m (°C)
**4**	F21T	2.0 µM	13.19
	FPu18T	2.0 µM	19.15
	F32T+H20M	2.0 µM	1.18
**5**	F21T	2.0 µM	12.95
	FPu18T	2.0 µM	16.66
	F32T+H20M	2.0 µM	1.34
**6**	F21T	2.0 µM	20.07
	FPu18T	2.0 µM	23.01
	F32T+H20M	2.0 µM	0.78

Supplementary Table 10: Acute toxicity of complex 6 in mice

Dose (mg/kg)	No. of animals	No. of death mice					Total death	Death (%) on day 14
		One day	Two day	Three day	Four day	Five-fourteen day		
15	10	7	1	1	0	0	9	90
12	10	3	1	1	0	0	4	40
9.6	10	0	1	0	0	0	1	10
7.8	10	0	0	0	0	0	0	0
Vehicle	10	0	0	0	0	0	0	0

Supplementary Table 11: The tumor volume in treated and non-treated mice from the date of surgery to the study end point in the BEL-7404 xenograft model

Group	Tumor volume (mm^3)		TGI (%)
	(start)	(end)	
Control	76.06±36.40	1178.54±189.16	-
**6 (4.0 mg/kg)**	75.81±19.95	782.94±73.43	35.9[a]
**6 (8.0 mg/kg)**	73.70±16.66	659.96±253.88	46.8[a]
**5-Fu (20.0 mg/kg)**	75.95±25.57	576.17±297.34	54.6[a]

A mean p<0.01, p *vs* vehicle control.

Supplementary Table 12: Average body weight in treated and non-treated mice from the date of surgery to the study end point in the BEL-7404 xenogfart model

Group	Body weight (g)		RBW (%)
	(start)	(end)	(end)
Control	19.2±0.9	18.4±0.9	95.7±3.7
**6 (4.0 mg/kg)**	19.5±1.6	17.2±1.7	87.9±4.2
**6 (8.0 mg/kg)**	19.5±1.2	17.4±0.8	89.2±2.5
**5-Fu (20.0 mg/kg)**	19.9±1.1	14.1±1.3	71.9±4.6

Supplementary Table 13: Data summary of BEL-7404 tumor growth inhibition

Group	Average tumor weight(mean ± SD g)	Inhibition of tumor growth(%)
Control	0.86±0.14	-
**6 (4.0 mg/kg)**	0.67±0.08	22.4[b]
**6 (8.0 mg/kg)**	0.51±0.19	41.2[a]
**5-Fu (20.0 mg/kg)**	0.47±0.20	45.1[a]

A mean p<0.01, b mean p<0.05, p *vs* control.

Supplementary Table 14: The tumor volume in treated and non-treated mice from the date of surgery to the study end point in the BEL-7402 xenograft model

Group	Tumor volume (mm^3)		TGI (%)
	(Start)	(End)	
Control	76.4±30.7	926.5±397.4	-
**6 (4.0 mg/kg)**	78.7±58.4	648.8±242.1	32.9
**6 (8.0 mg/kg)**	79.4±28.4	521.0±319.9	47.1[b]
Cisplatin (2.0 mg/kg)	78.6±37.7	329.9±155.2	69.8[a]

A mean $p<0.01$, b mean $p<0.05$, *p vs* vehicle control.

Supplementary Table 15: Average body weight in treated and non-treated mice from the date of surgery to the study end point in the BEL-7402 xenogfart model

Group	Body weight (g)		RBW (%)
	(Start)	(End)	(End)
Control	19.1±0.8	22.2±1.0	116.2±5.6
**6 (4.0 mg/kg)**	19.1±1.6	22.3±1.3	117.7±6.3
**6 (8.0 mg/kg)**	20.2±0.7	23.4±1.4	115.5±4.6
Cisplatin (2.0 mg/kg)	19.8±1.3	18.4±2.1	93.1±4.3

Supplementary Table 16: Data summary of BEL-7402 tumor growth inhibition

Group	Average tumor weight(mean ± SD g)	Inhibition of tumor growth(%)
Control	1.19±0.75	-
**6 (4.0 mg/kg)**	0.84±0.39	29.4
**6 (8.0 mg/kg)**	0.67±0.46	43.7[b]
Cisplatin (2.0 mg/kg)	0.38±0.18	68.4[a]

A mean $p<0.01$, b mean $p<0.05$, *p vs* control.

Supplementary Table 17: Changes in relative expression for tumor metastasis genes in the BEL-7404 cells after treated with 6. The table lists genes that exhibit a difference in expression in the BEL-7404 cells sample when compared to control. The raw threshold cycle (Ct) values seen in the samples are also listed for comparison.

See Supplementary File 2
