# Supplementary material for: Chiral platinum (II)-4-(2,3-dihydroxypropyl)- formamide oxo-aporphine (FOA) complexes promote tumor cells apoptosis by directly targeting G-quadruplex DNA in vitro and in vivo
Source: Oncotarget. 2017 Jun 28;8(37):61982–97. doi: 10.18632/oncotarget.18778 (PMC5617480; doi:10.18632/oncotarget.18778)
Supplement: Supplementary file 2 [file oncotarget-08-61982-s002.docx]

**Supplementary Table 1: Abbreviations, cell lines and DNA oligomers of used in this work**

| MTT | 3-(4,5-dimethylthiazol-2-yl)-2,5-diphenyltetrazolium bromide |
| --- | --- |
| TBS | pH 7.35, 10 mM Tris-KCl-HCl buffer solution, containing 100 mM KCl |
| TO | thiazole orange |
| SD | standard deviation |
| G4 | G-quadruplex |
| T_m_ | melting temperature |
| FRET | fluorescence resonance energy transfer |
| FID | fluorescent intercalator displacement |
| PI | propidium iodide |
| TAMRA | 6-carboxytetramethylrhodamine |
| PBS | phosphate buffered saline |
| TRF | the duplex TTAGGG repeat-binding factors (such as TRF1 and TRF2) |
| FAM | 6-carboxyfluorescein |
| hTERT | human telomerase reverse transcriptase |
| 53BP1 | p53 binding protein 1 |
| RT-PCR | reverse transcription-polymerase chain reaction |
| Pu27 | 5′-TGGGGAGGGTGGGGAGGGTGGGGAAGG-3′ |
| c-kit-2 | 5′-CGGGCGGGCGCTAGGGAGGGT-3′ |
| c-kit-1 | 5′-CGGGCGGGCACGAGGGAGGGT-3′ |
| HTG21 | 5′-GGGTTAGGGTTAGGGTTAGGG-3′ |
| ds26 | 5′-CAATCGGATCGAATTCGATCCGATTG-3′ |
| Pu22 | 5′-TGAGGGTGGGTAGGGTGGGTAA-3′ |
| F21T | 5′-FAM-GGGCTAGGGCTAGGGCTAGGG-TAMRA-3′ |
| FMidG4T | 5′-FAM-CGGGCGCGGGAGGAAGGGGGCGGGAGC-TAMRA-3′ |
| FPu18T | 5′-FAM-AGGGTGGGGAGGGTGGGG-TAMRA-3′ |
| H20M | 5′-GCCAGTTCTTGAATGTAGAG-3′ |
| F32T | 5′-FAM-CCGCATCTCTACATTCAAGAACTGGCATGCGG-TAMRA-3′ |
| hTERT | Cx: 5′-CATCCACATAGAGGCCACCACGT-3′  Ts: 5′-TGGTCTCCACGAGCCTCCGAGCG-3′ |
| c-myc | Cx: 5′-GTGGCACCTCTTGAGGACCT-3′  Ts: 5′-TGGTGCTCCATGAGGAGACA-3′ |
| GAPDH | Cx: 5′-CGGAAGGCCATGCCT GTCAG-3′  Ts: 5′-GCCTCTTGCACGACCAACTG-3′ |
| Hep-G2 | the hepatoblastoma cell line |
| MGC80-3 | the human gastric adenocarcinoma cell line |
| A549 | the human lung cancer cell line |
| BEL-7404 | the human hepatocellular carcinoma cell line |
| HeLa | the human cervical cancer cell line |
| HL-7702 | an human normal hepatocytes cell line |
