# Supplementary material for: Chiral platinum (II)-4-(2,3-dihydroxypropyl)- formamide oxo-aporphine (FOA) complexes promote tumor cells apoptosis by directly targeting G-quadruplex DNA in vitro and in vivo
Source: Oncotarget. 2017 Jun 28;8(37):61982–97. doi: 10.18632/oncotarget.18778 (PMC5617480; doi:10.18632/oncotarget.18778)
Supplement: Supplementary file 3 [file oncotarget-08-61982-s003.docx]

**Supplementary Table 17:** Changes in relative expression for tumor metastasis genes in the BEL-7404 cells after treated with **6**. The table lists genes that exhibit a difference in expression in the BEL-7404 cells sample when compared to control. The raw threshold cycle (Ct) values seen in the samples are also listed for comparison.

| **Gene** | **Fold Change** | **p value** | | **Fold Up-or**  **Down-Regulation** | | | **Comments** |  |
| --- | --- | --- | --- | --- | --- | --- | --- | --- |
| ABL1 | 0.80 | 0.374160 | | -1.25 | | | OKAY |  |
| ACD | 1.16 | 0.348494 | | 1.16 | | | OKAY |  |
| AKT1 | 0.77 | 0.440171 | | -1.30 | | | OKAY |  |
| ATM | 1.08 | 0.384739 | | 1.08 | | | OKAY |  |
| ATP5C1 | 1.19 | 0.187322 | | 1.19 | | | OKAY |  |
| BCL2 | 0.76 | 0.105189 | | -1.32 | | | OKAY |  |
| BLM | 1.21 | 0.136819 | | 1.21 | | | OKAY |  |
| CDK2 | 0.80 | **0.010476** | | -1.24 | | | OKAY |  |
| CHEK1 | 1.13 | 0.295316 | | 1.13 | | | OKAY |  |
| CHEK2 | 1.23 | 0.065000 | | 1.23 | | | OKAY |  |
| DCLRE1B | 0.81 | 0.164222 | | -1.24 | | | OKAY |  |
| DCLRE1C | 1.20 | 0.505210 | | 1.20 | | | OKAY |  |
| DKC1 | 1.14 | 0.244136 | | 1.14 | | | OKAY |  |
| EGF | 1.11 | 0.678199 | | 1.11 | | | B |  |
| EME1 | 1.26 | 0.112879 | | 1.26 | | | OKAY |  |
| ERCC1 | 1.00 | 0.928958 | | -1.00 | | | OKAY |  |
| ERCC4 | 1.22 | 0.096950 | | 1.22 | | | OKAY |  |
| GAR1 | 0.95 | 0.652073 | | -1.05 | | | OKAY |  |
| HAT1 | 1.13 | 0.513779 | | 1.13 | | | OKAY |  |
| HNRNPA2B1 | 1.06 | 0.545580 | | 1.06 | | | OKAY |  |
| HNRNPD | 0.95 | 0.691806 | | -1.06 | | | OKAY |  |
| HSP90AA1 | 1.35 | **0.023426** | | 1.35 | | | OKAY |  |
| HSPA1L | 1.65 | **0.011772** | | 1.65 | | | OKAY |  |
| IGF1 | **0.02** | **0.000089** | | **-57.88** | | | OKAY |  |
| KRAS | 0.88 | 0.107118 | | -1.13 | | | OKAY |  |
| KRIT1 | 1.12 | 0.282693 | | 1.12 | | | OKAY |  |
| MEN1 | 1.14 | 0.212944 | | 1.14 | | | OKAY |  |
| MRE11A | 1.41 | **0.000677** | | 1.41 | | | OKAY |  |
| MSH2 | 0.90 | **0.039522** | | -1.11 | | | OKAY |  |
| MSH3 | 0.66 | **0.007063** | | **-1.51** | | | OKAY |  |
| MUS81 | 1.55 | **0.003241** | | **1.55** | | | OKAY |  |
| MYC | 0.75 | **0.040987** | | -1.33 | | | OKAY |  |
| NBN | 0.79 | 0.107711 | | -1.27 | | | OKAY |  |
| NCL | 1.04 | 0.626324 | | 1.04 | | | OKAY |  |
| NHP2 | 1.46 | **0.012661** | | 1.46 | | | OKAY |  |
| NOP10 | 0.82 | | 0.233491 | | -1.22 | OKAY | | |
| OBFC1 | 1.32 | | **0.024465** | | 1.32 | OKAY | | |
| PARP1 | 1.02 | | 0.803710 | | 1.02 | OKAY | | |
| PAX8 | **9.91** | | **0.009468** | | **9.91** | OKAY | | |
| PIF1 | 0.84 | | 0.171666 | | -1.19 | OKAY | | |
| PINX1 | 1.13 | | 0.375175 | | 1.13 | OKAY | | |
| PLK1 | 0.72 | | **0.002509** | | -1.39 | OKAY | | |
| POT1 | 0.87 | | 0.257171 | | -1.14 | OKAY | | |
| PPARG | 1.08 | | 0.334445 | | 1.08 | OKAY | | |
| PPP2R1A | 0.88 | | 0.328164 | | -1.13 | OKAY | | |
| PPP2R1B | 0.68 | | **0.003468** | | -1.46 | OKAY | | |
| PRKCA | 1.12 | | **0.038877** | | 1.12 | OKAY | | |
| PRKCB | **0.48** | | 0.266044 | | **-2.06** | B | | |
| PRKDC | 0.83 | | **0.022103** | | -1.20 | OKAY | | |
| PTGES3 | 1.07 | | 0.404900 | | 1.07 | OKAY | | |
| PURA | 1.07 | | 0.598919 | | 1.07 | OKAY | | |
| RAD17 | 1.11 | | 0.256885 | | 1.11 | OKAY | | |
| RAD50 | 1.44 | | 0.104899 | | 1.44 | OKAY | | |
| RAP1A | 0.90 | | 0.406713 | | -1.11 | OKAY | | |
| RAPGEF1 | 0.97 | | 0.818476 | | -1.03 | OKAY | | |
| RASSF1 | 1.26 | | 0.086582 | | 1.26 | OKAY | | |
| RB1 | 0.59 | | **0.002241** | | **-1.70** | OKAY | | |
| RFC1 | 1.33 | | 0.120362 | | 1.33 | OKAY | | |
| RIF1 | 1.67 | | **0.010746** | | **1.67** | OKAY | | |
| RTEL1 | **0.39** | | **0.023338** | | **-2.58** | OKAY | | |
| SART1 | 1.50 | | **0.030945** | | 1.50 | OKAY | | |
| SIRT2 | 0.84 | | 0.116054 | | -1.19 | OKAY | | |
| SIRT6 | 1.89 | | **0.000463** | | **1.89** | OKAY | | |
| SLX4 | 0.87 | | 0.222345 | | -1.15 | OKAY | | |
| SMAD3 | 0.65 | | 0.183275 | | -1.55 | B | | |
| SMG6 | 1.05 | | 0.536653 | | 1.05 | OKAY | | |
| SP1 | 0.70 | | **0.000432** | | -1.43 | OKAY | | |
| SSB | 1.14 | | 0.360112 | | 1.14 | OKAY | | |
| SUN1 | 1.04 | | 0.476789 | | 1.04 | OKAY | | |
| TEP1 | 1.73 | | **0.004042** | | **1.73** | OKAY | | |
| TERF1 | 1.39 | | 0.088766 | | 1.39 | OKAY | | |
| TERF2 | 0.99 | | 0.949892 | | -1.01 | OKAY | | |
| TERF2IP | 1.47 | | **0.007190** | | 1.47 | OKAY | | |
| TERT | 0.89 | | 0.274269 | | -1.12 | B | | |
| TGFB1 | 1.46 | | **0.004143** | | 1.46 | OKAY | | |
| TINF2 | 1.14 | | **0.042999** | | 1.14 | OKAY | | |
| TNKS | 1.12 | | **0.023395** | | 1.12 | OKAY | | |
| TNKS2 | 1.02 | | 0.853284 | | 1.02 | OKAY | | |
| TP53 | 1.45 | | **0.003933** | | 1.45 | OKAY | | |
| TP53BP1 | 1.18 | | 0.072980 | | 1.18 | OKAY | | |
| TPP1 | 1.07 | | 0.369829 | | 1.07 | OKAY | | |
| WRAP53 | 0.94 | | 0.863951 | | -1.06 | OKAY | | |
| XRCC5 | 0.76 | | 0.178626 | | -1.32 | OKAY | | |
| XRCC6 | 1.15 | | 0.569369 | | 1.15 | OKAY | | |
| ACTB | 0.76 | | **0.001515** | | -1.32 | OKAY | | |
| B2M | 1.15 | | 0.204904 | | 1.15 | OKAY | | |
| GAPDH | 0.97 | | 0.541419 | | -1.04 | OKAY | | |
| HPRT1 | 1.14 | | **0.017556** | | 1.14 | OKAY | | |
| RPLP0 | 1.05 | | 0.392150 | | 1.05 | OKAY | | |

**Legend:** **Fold-Change** (2^-Delta Delta Ct^) is the normalized gene expression (2^-Delta Ct^) in the Test Sample divided the normalized gene expression (2^-Delta Ct^) in the Control Sample.

**Fold-Regulation** represents fold-change results in a biologically meaningful way. Fold-change values greater than one indicate a positive- or an up-regulation, and the fold-regulation is equal to the fold-change

Fold-change values less than one indicate a negative or down-regulation, and the fold-regulation is the negative inverse of the fold-change.

Fold-change and fold-regulation values greater than 2 are indicated in red; fold-change values less than 0.5 and fold-regulation values less than -2 are indicated in blue.

**p-values:** The p values are calculated based on a Student’s t-test of the replicate 2^-Delta Ct^ values for each gene in the control group and treatment groups, and p values less than 0.05 are indicated in red.

**Comments:** A: This gene’s average threshold cycle is relatively high (>30) in either the control or the test sample, and is reasonably low in the other sample (<30).

These data mean that the gene’s expression is relatively low in one sample and reasonably detected in the other sample suggesting that the actual fold-change value is at least as large as the calculated and reported fold-change result. This fold-change result may also have greater variations if p value >0.05; therefore, it is important to have a sufficient number of biological replicates to validate the result for this gene. B: This gene’s average threshold cycle is relatively high (>30), meaning that its relative expression level is low, in both control and test samples, and the p-value for the fold-change is either unavailable or relatively high (p>0.05). This fold-change result may also have greater variations; therefore, it is important to have a sufficient number of biological replicates to validate the result for this gene. C: This gene’s average threshold cycle is either not determined or greater than the defined cut-off (deault 35), in both samples meaning that

its expression was undetected, making this fold-change result erroneous and un-interpretable.
